# Improved reference genome of the arboviral vector *Aedes albopictus*

**DOI:** 10.1186/s13059-020-02141-w

**Published:** 2020-08-26

**Authors:** Umberto Palatini, Reem A. Masri, Luciano V. Cosme, Sergey Koren, Françoise Thibaud-Nissen, James K. Biedler, Flavia Krsticevic, J. Spencer Johnston, Rebecca Halbach, Jacob E. Crawford, Igor Antoshechkin, Anna-Bella Failloux, Elisa Pischedda, Michele Marconcini, Jay Ghurye, Arang Rhie, Atashi Sharma, Dmitry A. Karagodin, Jeremy Jenrette, Stephanie Gamez, Pascal Miesen, Patrick Masterson, Adalgisa Caccone, Maria V. Sharakhova, Zhijian Tu, Philippos A. Papathanos, Ronald P. Van Rij, Omar S. Akbari, Jeffrey Powell, Adam M. Phillippy, Mariangela Bonizzoni

**Affiliations:** 1 Department of Biology and Biotechnology, University of Pavia, Pavia, 27100 Italy; 2 Department of Entomology and the Fralin Life Science Institute, Virginia Polytechnic and State University, Blacksburg, VA 24061 USA; 3 Department of Ecology and Evolutionary Biology, Yale University, New Haven, CT 06511-8934 USA; 4 Genome Informatics Section, Computational and Statistical Genomics Branch, National Human Genome Research Institute, National Institutes of Health, Bethesda, 20892-2152 MD USA; 5 National Center for Biotechnology Information, National Library of Medicine, National Institutes of Health, Bethesda, 20894 MD USA; 6 Department of Entomology, Robert H Smith Faculty of Agriculture, Food and Environment, The Hebrew University of Jerusalem, 7610001 Rehovot, Israel; 7 Department of Entomology, Texas A&M University, College Station, TX 77843 USA; 8 Department of Medical Microbiology, Radboud University Medical Center, Radboud Institute for Molecular Life Sciences, P.O. Box 9101, 6500 HB Nijmegen, The Netherlands; 9 Verily Life Sciences, South San Francisco, 94080 CA USA; 10 Division of Biology and Biological Engineering, California Institute of Technology, Pasadena, CA 91125 USA; 11 Department of Virology, Arbovirus and Insect Vectors Units, Institut Pasteur, Paris, 75015 France; 12 Laboratory of Evolutionary Genomics of Insects, The Federal Research Center Institute of Cytology and Genetics, Siberian Branch of the Russian Academy of Sciences, Novosibirsk, 630090 Russia; 13 Laboratory of Ecology, Genetics and Environment Protection, Tomsk State University, Tomsk, 634041 Russia; 14 Division of Biological Sciences, University of California, San Diego, La Jolla, CA 92093-0349 USA

**Keywords:** *Ae. albopictus*, Genome, miRNAs, piRNA clusters, Viral integrations, Immunity, Sex locus, Population differentiation, Developmental transcriptome

## Abstract

**Background:**

The Asian tiger mosquito *Aedes albopictus* is globally expanding and has become the main vector for human arboviruses in Europe. With limited antiviral drugs and vaccines available, vector control is the primary approach to prevent mosquito-borne diseases. A reliable and accurate DNA sequence of the *Ae. albopictus* genome is essential to develop new approaches that involve genetic manipulation of mosquitoes.

**Results:**

We use long-read sequencing methods and modern scaffolding techniques (PacBio, 10X, and Hi-C) to produce AalbF2, a dramatically improved assembly of the *Ae. albopictus* genome. AalbF2 reveals widespread viral insertions, novel microRNAs and piRNA clusters, the sex-determining locus, and new immunity genes, and enables genome-wide studies of geographically diverse *Ae. albopictus* populations and analyses of the developmental and stage-dependent network of expression data. Additionally, we build the first physical map for this species with 75% of the assembled genome anchored to the chromosomes.

**Conclusion:**

The AalbF2 genome assembly represents the most up-to-date collective knowledge of the *Ae. albopictus* genome. These resources represent a foundation to improve understanding of the adaptation potential and the epidemiological relevance of this species and foster the development of innovative control measures.

## Background

Climate change, urbanization, and increased international mobility are predicted to further increase the spreading of the highly invasive mosquito *Aedes albopictus* and severely exacerbate the risk and burden of *Aedes*-transmitted human pathogens, *in primis* dengue, Zika, and chikungunya viruses, but also the veterinary-relevant parasite *Dirofilaria immitis* [1, 2]. As a consequence, nearly a billion people could face their first exposure to arboviral transmission within the next century especially in subtropical and temperate regions of the world, including Europe [2].

The initial genome assembly of *Ae. albopictus* (AaloF1) from the Chinese Foshan strain represented a fundamental achievement for the genetic characterization of this mosquito [3]. From this analysis, based solely on the assembly of short DNA sequence reads, the genome of *Ae. albopictus* appears to be the largest mosquito genome sequenced to date (1.9 Gb). However, due to very high levels of repetitive DNA and reliance on short-read sequencing, AaloF1 remains highly fragmented with more than 150,000 scaffolds, limiting its utility.

## Results

**Fig. 1:**
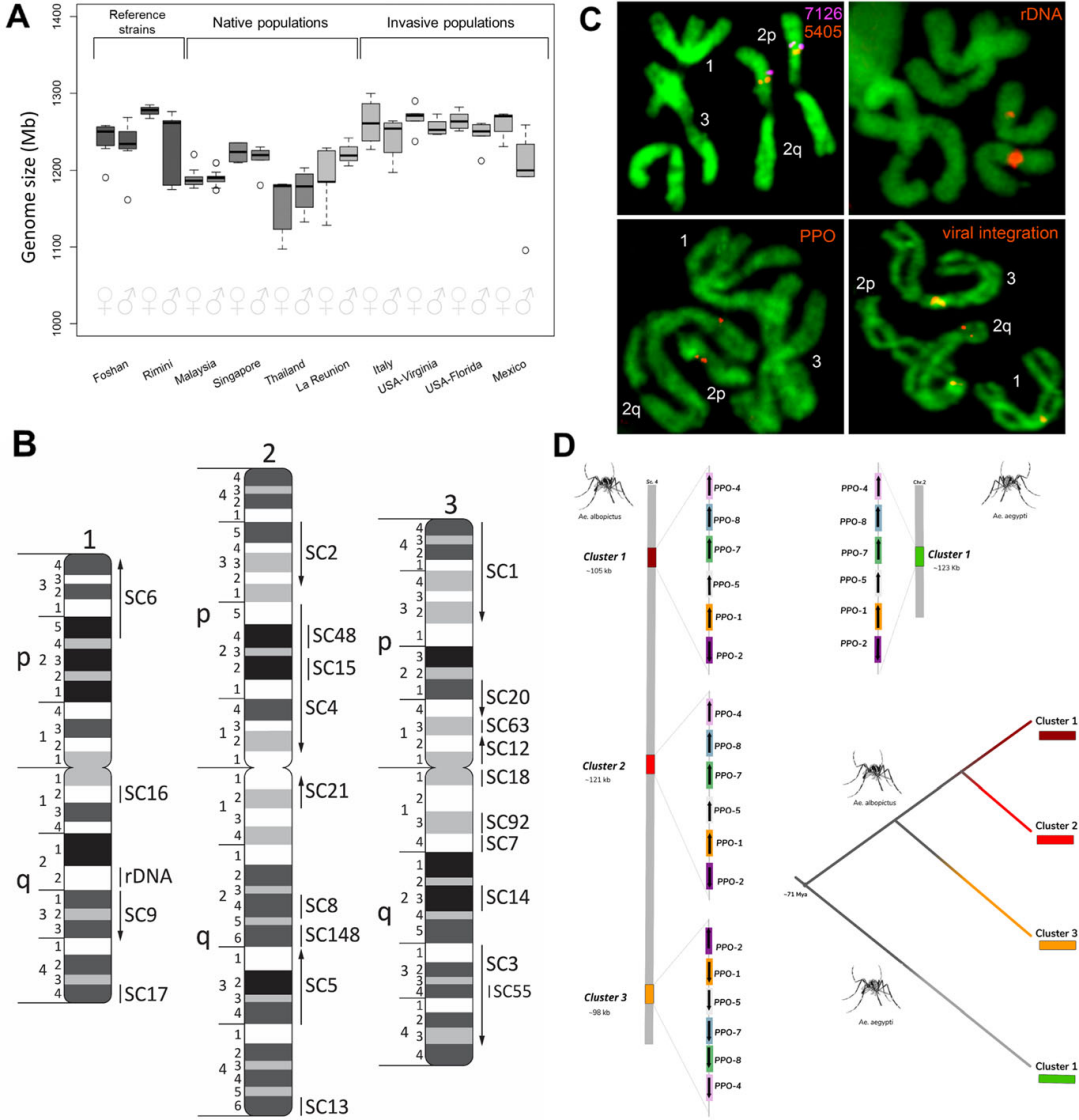
Size of the *Aedes albopictus* genome and physical map. **a** Cytofluorimetric-based estimates of the genome size of *Ae. albopictus* strains, including Foshan and Rimini from which genome assemblies were derived based on short-read Illumina sequencing [3, 4] and *Ae. albopictus* wild-collected samples from the native home range (Malaysia, Singapore, Thailand), an old-colonized region (La Reunion), and newly invaded areas (the USA, Mexico, Italy). The *Ae. albopictus* genome size is estimated to be in the range of 1095–1299 Mb, comparable or slightly larger than that of *Ae. aegypti* (1066–1309 Mb) [5]. **b** Physical genome map of *Ae. albopictus* based on 50 DNA probes hybridized in situ to mitotic chromosomes. Chromosomes and chromosome arms are indicated by numbers 1, 2, and 3 and letters p and q, respectively. Chromosome divisions and subdivisions are shown on the left sides of the idiograms. Scaffolds are indicated by arrows or lines. Arrows indicate orientations of the scaffolds. SC stands for scaffold; rDNA stands for ribosomal locus. **c** Examples of fluorescence in situ hybridization. Chromosomal locations of transcripts XM_019675405 and XM_020077126 from scaffolds 4 and 48, respectively; rDNA, polyphenol oxidase (PPO) gene clusters, and the largest viral integration in the genome (Canu-Flavi19) are demonstrated. Transcripts are indicated on a figure by the last four digits of their accession numbers. **d** Schematic illustration of chromosomal locations of PPO cluster triplication in the new assembly of *Ae. albopictus* (GCF_006496715.1). Comparative genomics analysis of the synteny in the AaegL5 genome reveals an array of 6 genes localized in a region of 123.44 kb at chromosome 2 (2:199,230,485-199,353,929) and was locally duplicated twice in *Ae. albopictus*, resulting in 18 PPO genes. PPO gene cluster array of chromosome 2 of *Ae. aegypti* includes AAEL015116 (PPO1), AAEL015113 (PPO2), AAEL013492 (PPO5), AAEL013493 (PPO7), AAEL013501 (PPO4), and AAEL013496 (PP08)

Using a cytofluorimetric approach, we estimated the genome length of *Ae. albopictus* to be similar to that of *Ae. aegypti*, between 1.190–1.275 Gb, across populations from the native home range (Thailand, Malaysia, Singapore), old-colonized regions (La Reunion Island), and recently invaded areas (Italy, the USA, and Mexico) (Fig. 1a).

To foster continuity, we chose to use the Foshan strain for further genome study. After six consecutive rounds of single sister-brother matings, we extracted high-molecular weight DNA from forty sibling mosquitoes. We then generated approximately 82 Gb of PacBio single-molecule long reads with a mean read length of 10 kb and an N50 length of 18 kb (N50 length: half of the data comprises sequences of this length or longer). Additionally, we prepared a Hi-C proximity ligation library from ten adult mosquitoes and collected 135 Gb of Illumina reads. We assembled the long-read PacBio data with Canu [6] and polished the resulting contigs with Arrow (https://www.pacb.com/products-and-services/analytical-software/smrt-analysis/) using the raw PacBio signal data. This initial assembly totaled 5.17 Gb, far exceeding the expected haploid genome size (~ 1.25 Gb), suggesting the presence of alleles that failed to collapse in the assembly. We hypothesized that this was due to high levels of heterozygosity in the pool of sequenced mosquitoes, resulting in multiple allelic variants assembled separately, as has been previously noted in long-read assemblies [7]. To partition this initial assembly into primary and alternative contig sets, we analyzed contig alignments and depth of coverage with Purge Haplotigs [7] along with BUSCO single-copy orthologs [8] to determine which contigs were likely to be redundant and should be designated as alternative alleles. Haplotig purging reduced the size of the primary assembly by nearly half to 2.54 Gb, which was then scaffolded via the Hi-C data using SALSA2 [9].

The final primary assembly, which we call AalbF2, consists of 2197 scaffolds with an N50 length of 55.7 Mb (Additional file 2: Table S1). This represents a continuity increase of two orders of magnitude compared to AaloF1 scaffold N50s of 201 kb [3]. This significant increase in continuity provides a more complete view of the genomic organization of *Ae. albopictus* and allows for a more accurate annotation of gene structures.

Analyses of single-copy orthologs via BUSCO [8] in AalbF2 showed an 8.3% increase in the percentage of complete, single-copy BUSCOs with respect to AaloF1 (Additional file 2: Table S1). AalbF2 has a BUSCO completeness of 93.2%, with an estimated 14.0% duplication. Additionally, using Barrnap (https://github.com/tseemann/barrnap), the number of ribosomal RNA gene sequences was estimated to be 484 in AalbF2 (compared to 22 in AaloF1), a value close to the number (430) independently estimated from an *Ae. albopictus* haploid genome [10]. The rate of alignments of DNA and RNA sequencing data from published resources [11–13] and the percentage of properly paired reads were also analyzed and confirmed the quality and continuity of AalbF2 (Additional file 2: Table S1). The higher continuity of AalbF2 is also shown by the annotation of transposable elements (TE), which amount to 55.03% of the genome size, a value comparable to that of the most recent assembly of the *Ae. aegypti* genome, AaegL5 (Additional file 2: Table S2).

The original, unfiltered, and unsplit assembly (main and alternative scaffolds) had a BUSCO completeness of 97.6% with 81.8% duplication, indicating that the majority of genes were represented in the combined assembly by more than one allele. Despite promising improvements to long-read sequencing methods that have enabled genome assembly from a single *Anopheles coluzzii* mosquito [14], the larger genome size of *Aedes* spp. mosquitoes (i.e., 1.2 Gb vs. 280 Mb for *An. coluzzii*) required pooling of heterozygous individuals and the necessity of removing haplotypic duplications prior to the creation of haploid reference scaffolds [5].

A total of 26,856 protein-coding sequences were predicted in AalbF2 through the NCBI Eukaryotic Genome Annotation Pipeline (https://www.ncbi.nlm.nih.gov/genome/annotation euk/process/). To help distinguish between artifacts and genuine gene duplications, which are resistant to proper assembly, and mitigating the heterozygosity effect from the original pooled DNA, we developed a pipeline based on the assumptions that selection acts mainly on the coding sequence of a gene and that homology between highly related paralogs drops in the flanking untranslated sequences (Additional file 1: Fig. S1). To perform the analysis, we compared 500 bp or 1000 bp of the flanking regions at the 5′ and 3′ ends of all candidate gene duplicate with an all-against-all BLASTn search with an *e* value of 1 × 10^−40^ for each flanking region. We found 1329 (8.05% of total) genes with high similarity within 500 bp of their 5′ and 3′ flanking regions mapping on 452 of the 2196 scaffolds (Additional file 3). When we considered the extended 1000-bp regions, the number of candidates duplicated was lower (808 mapping on 300 scaffolds, 4.89% of total). Most of these artifacts involved a single duplicated gene (twins), and the number decreased with increasing copies. A list of gene duplications that are likely to be artifacts of the assembly is available for future reference in Additional file 3.

A significant improvement of AalbF2 is that more than 50% of the genome assembly is contained within the 13 largest scaffolds (e.g., L50 = 13; L75 = 58, Additional file 1: Fig. S2, Additional file 2: Table S1). We developed a physical genome map of the *Ae. albopictus* genome using in situ hybridization on mitotic chromosomes covering 57% of the genome assembly by targeting twenty of the largest genomic scaffolds and three minor scaffolds (Additional file 2: Table S3, Fig. 1b). A total of 4, 9, and 10 scaffolds were assigned to chromosomes 1, 2, and 3, respectively. Positions of the transcript from scaffolds 15, 48, and 55 hybridized to places already covered with other large scaffolds. The positions of all tested transcripts were consistent with their positions in the *Ae. aegypti* genome, which is assembled into chromosome-size scaffolds, providing an independent confirmation of the accuracy of the in situ hybridization results [5]. Based on probe mapping to the *Ae. aegypti* genome and homology between the *Ae. aegypti* and the *Ae. albopictus* chromosomes (Additional file 1: Fig. S2), we bioinformatically assigned the 58 longest scaffolds covering 75% of the genome to *Ae. albopictus* chromosomes (Additional file 2: Table S4).

Cytogenetic comparison (Table 1) between *Ae. albopictus* and *Ae. aegypti* demonstrated that the total chromosome length is 4.9 μm or 16.4% longer in *Ae. albopictus* (*P* < 0.0001), which suggests a slightly larger genome size in this species, as also suggested by cytofluorimetry. Chromosome proportions, such as relative chromosome and arm lengths, between the two species were also different. In *Ae. albopictus*, “chromosome 1” was shorter but chromosome 2 was longer relative to *Ae. aegypti*. Besides positioning and orienting the largest scaffolds, we physically mapped the18S rDNA and other genomic features (e.g., viral integrations and representative immunity genes) described below (Fig. 1c, d). The 18S rDNA mapped in the region of the secondary constriction in region 1q22. The intensity of the signal significantly varied among chromosomes from individual mosquitoes suggesting variations in numbers of ribosomal genes.

**Table 1:** Comparison between *Ae. aegypti* and *Ae. albopictus* mitotic chromosomes

Chromosome length/proportions	***Ae. albopictus***	***Ae. aegypti***
**Chromosome 1**		
Average length, μm	8	7.1
Relative length (%; *P* value*)	26.8; *P* < 0.0001	28.4
Centromeric index (%; *P* value)	46.7; *P* = 0.0044	46.9
**Chromosome 2**		
Average length, μm	11.7	9.5
Relative length (%; *P* value)	39.1; *P* < 0.0001	38
Centromeric index (%; *P* value)	46.9; *P* = 0.0051	48.6
**Chromosome 3**		
Average length, μm	10.2	8.4
Relative length (%; *P* value)	34.1; *P* < 0.0001	33.6
Centromeric index (%; *P* value)	47.2; *P* = 0.0079	47.4

*P value indicates significant difference in relative length and centromeric index between *Ae. albopictus* and *Ae. aegypti* chromosomes

### The landscape of endogenous viral elements

The genome of *Ae. albopictus* harbors hundreds of integrated sequences from nonretroviral RNA viruses, called nonretroviral endogenous viral elements (nrEVE) or nonretroviral integrated RNA virus sequences (NIRVS) (Palatini et al.). Taking advantage of the contiguity of AalbF2 and using a viral database composed of 1563 viral species (Additional file 4), we revised the annotation of nrEVEs, while also providing correspondence with viral integrations previously annotated in AaloF1 (Additional file 5, Additional file 2: Table S5). Additionally, we used the identified viral integrations to screen the alternative assembly (NCBI accession GCA_006496715.1) and found alternative nrEVE alleles (Additional file 2: Table S6), confirming that the haplotig purging applied to the initial assembly effectively moved haplotypic variants into the alternative assembly.

**Fig. 2:**
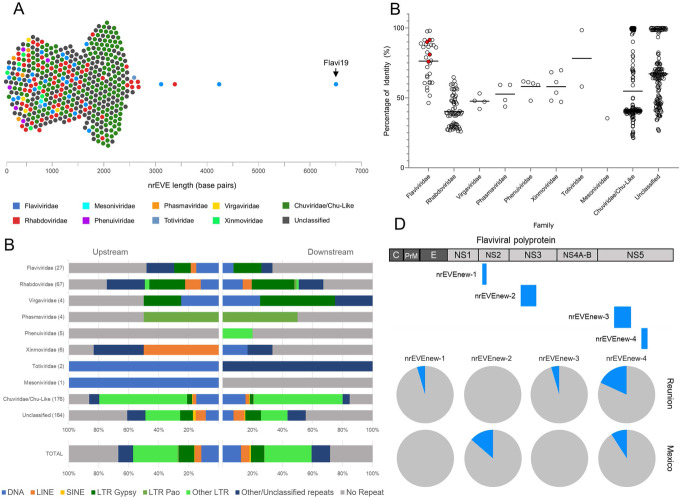
Atlas of viral integration in the *Aedes albopictus* genome. **a** Bee swarm plot showing viral integrations identified in the *Ae. albopictus* genome (AalbF2). Each dot is a viral integration, plotted according to its length and color-coded based on its viral origin. nrEVEs range in length from 131 to 6593 nt, with an average of 1289 nt. Arrow points to Canu-Flavi19, the longest nrEVE. **b** Scatter plot representing the amino acid identity of each nrEVE and its best hit retrieved by blastx searches against NR database grouped by viral family. The average is shown by a line. Red dots are the novel viral integrations discovered in wild-caught mosquitoes. **c** Bar plots showing the type of the closest transposable element, which was identified upstream and downstream each nrEVE. Viral integrations are classified based on their viral origin as shown in **a**. **d** Scheme of the novel viral integrations identified in the genome of wild-collected mosquitoes with respect to a *Flavivirus* genome and their frequency occurrence in mosquitoes from Tampon (Reunion) and Tapachula (Mexico)

We confirmed that the majority of nrEVEs of *Ae. albopictus* genome have similarities to known insect-specific flaviviruses (ISFs) and rhabdoviruses (Fig. 2a, b), which tend to map less than 10 kb to each other, generating clusters of often rearranged or duplicated sequences (Additional file 1: Fig. S3), and are in tight association with transposable elements (TE), primarily Gypsy and Pao LTR (Fig. 2c). This association appears to be driven by the enrichment of LTR retrotransposons into piRNA clusters (Additional file 1: Fig. S3).

The largest viral integration (Canu-Flavi19) in AalbF2 reached 6593 bp and encompassed all of the structural proteins, the entire NS1 and NS2, and part of the NS3 and NS5 proteins of the 11,064 bp genome of *Aedes* flavivirus, with a 97,63 percentage of identity (Additional file 5). This viral integration was mapped by in situ hybridization to chromosome 2q close to the telomere, confirming it is integrated within the genome (Fig. 1b). Signals were also found in the centromeres of all three chromosomes, probably because these regions contain nrEVEs with sequence similarity to Canu-Flavi19 (Fig. 1b).

Using genome engineering approaches of both viral and mosquito genomes, selected nrEVEs were shown to exert antiviral activity with respect to cognate viral infections [15, 16]. These results suggest nrEVEs are heritable immunity sequences, which implies that their distribution patterns may differ across geographic populations depending on viral exposure [17–19]. To address whether viral integrations different from those annotated in AalbF2 can be characterized in wild-caught mosquitoes (hereafter called novel nrEVEs), we collected and sequenced the genomes of 24 adult females from Tapachula (Mexico) and Tampon (La Reunion island) where several arboviruses are endemic. By using Vy-Per [20] followed by ViR [21], we identified one and two novel viral integrations in samples from Tapachula and Tampon, respectively, plus a novel viral integration common to both populations (Fig. 2d). Two of these novel viral integrations (nrEVEnew-3 and nrEVEnew-4) have similarities to AeFV, one (nrEVEnew-2) to CFAV and the other (nrEVENew-1) to KRV (Fig. 2d). All these novel viral integrations were molecularly validated by designing specific PCR primers (Additional file 2: TableS7, Additional file 1: Fig. S3B). Novel viral integrations were more frequent in mosquitoes from Tampon than Tapachula (Fig. 2d). Additionally, two of the Tampon novel viral integrations had a 90% amino acid identity with AeFV and CFAV, respectively (compared to the 72% average identity for annotated Flavi-EVEs), suggesting recent integration events. This result correlates with the invasion history of *Ae. albopictus* out of its native home range in Asia. Before the aggressive global invasion of *Ae. albopictus*, which started roughly 50 years ago, *Ae. albopictus* had reached the islands of the Indian and Pacific Oceans from South East Asia in the eighteenth to early twentieth centuries [22]. Thus, mosquitoes from La Reunion Island are considered “old” and have maintained large populations [23, 24]. In contrast, *Ae. albopictus* was first detected in Tapachula in 2002, likely a secondary invasion from the USA or Italy [25, 26].

### Distribution and structure of piRNA clusters

PIWI-interacting RNAs (piRNAs) are mostly known for their role in immunity against TEs in the germline [27]. This is best studied in the model organism *Drosophila melanogaster*. However, in *Aedes* spp. mosquitoes, the piRNA pathway acquired additional functions in antiviral immunity and can use viral RNAs as a substrate for piRNA production [28]. Most piRNAs are derived from large genomic regions termed piRNA clusters. These clusters present a memory of past transposon invasions and confer immunity against these elements, as piRNAs processed from transposon remnants within clusters can target active transposons encoded elsewhere in the genome [27].

**Fig. 3:**
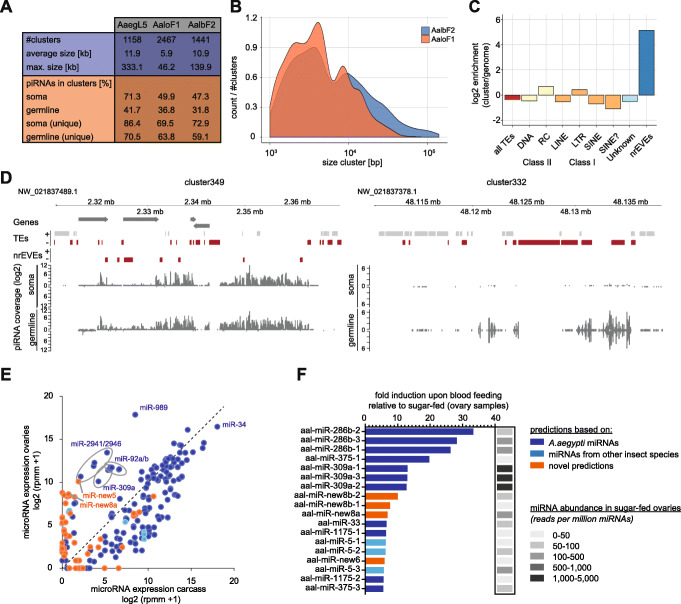
Small non-coding RNA annotation in AalbF2. **a** Summary statistics on annotated piRNA clusters using the genome assemblies for *Ae. aegypti* AaegL5 and *Ae. albopictus* AaloF1 or AalbF2. **b** Size distribution of piRNA clusters annotated with the old AaloF1 assembly or the most recent AalbF2 assembly. The density plot shows the number of clusters normalized to the total number of piRNA clusters. **c** Enrichment of repeat classes or nrEVEs in piRNA clusters compared to the whole genome. **d** log2 piRNA coverage on an exemplary uni-strand (left panel) or dual-strand (right panel) piRNA cluster (given as piRNAs per million mapped reads [rpm]). Annotated genes are indicated with arrows, repeat features, and nrEVEs with gray or red boxes for positive or negative strands, respectively. **e** miRNA abundance in *Ae. albopictus* carcass and ovary samples. Counts for individual miRNAs were normalized to the total number of miRNAs in each dataset and expressed as log2-transformed reads per million miRNAs (rpmm) + 1. The mean of two independent libraries for each condition is shown. The highly abundant miR-34 and selected miRNAs with high expression in the ovary, but not carcass, are indicated. **f** Fold induction of miRNA levels in blood-fed ovary samples compared to sugar-fed samples. The basal expression of each miRNA in the sugar-fed samples is indicated in gray scale. Only miRNAs with an induction ≥ 5-fold are shown. Color coding in **a** and **b** represents the basis for the miRNA prediction, as indicated

Using the preceding AaloF1 genome assembly, a previous study reported 643 clusters with a maximum length of maximum 10 kb [29]. However, piRNA clusters can span up to several hundred kilobases [30, 31]; therefore, a more continuous genome assembly can improve the annotation of these genomic regions. We used small RNA libraries generated from somatic tissues (female carcass) as well as germline tissues (ovaries) to annotate 1441 piRNA clusters with an average size of 10.911 kb (SD 634.885 kb; max: 139.92 kb) (Additional file 1: Fig. S4, Additional file 6), covering 0.62% of the genome. This is comparable to piRNA clusters annotated with the same approach in *Ae. aegypti* (Fig. 3a). In contrast, using the same annotation pipeline on the highly fragmented *Ae. albopictus* AaloF1 genome assembly, we recovered nearly twice as many (2467) but much smaller clusters (average size, 5.923 kb; SD, 306.239 kb; max, 64.225 kb) (Fig. 3a, b). Only a comparably small fraction (31.8% and 47.3%) of all piRNAs in the germline and soma, respectively, were included in piRNA clusters in AalbF2, while this fraction was nearly twice as large in *Ae. aegypti* (Fig. 3a). This is likely accounted for by the 14% of duplications still present in the assembly, leading to the exclusion of piRNA clusters without or with only very few uniquely mapping piRNAs; the presence of which was used as a criterium to annotate piRNA clusters. Consequently, when only considering unambiguously mapping piRNAs, the fraction of piRNAs included in clusters increases to 59.1% and 72.9% in germline and soma, respectively.

The vast majority of all clusters display piRNA expression biased towards one strand, and only approximately one fifth of all clusters were expressed from both strands (see exemplary clusters in Fig. 3c). Such dual strand clusters were mostly expressed in the germline (Additional file 1: Fig. S4). Interestingly, relative piRNA expression from clusters varied substantially between somatic and germline tissues, with some clusters showing a soma-dominant expression and others being predominantly expressed in the germline. Blood-feeding had little impact on cluster expression. Analysis of publicly available small RNA libraries derived from the widely used *Ae. albopictus* C6/36 and U4.4 cell lines showed piRNA production from both somatic and germline clusters (Additional file 1: Fig. S4).

While piRNA clusters are highly enriched with transposable elements in fruit flies [31], this is not the case in *Ae. aegypti* mosquitoes [32], even though their genomic transposon content is much higher. Comparably, only a minority of *Ae. albopictus* piRNAs were derived from repetitive elements [29], and piRNA clusters were slightly depleted of all repetitive sequences except for helitrons and LTR-retrotransposons (Fig. 3c). Interestingly, nrEVEs were enriched compared to the rest of the genome (Additional file 1: Fig. S3), and 138 out of 456 elements were overlapping with piRNA clusters, suggesting strong evolutionary pressure to integrate viral sequences into piRNA clusters and/or maintain nrEVEs in piRNA-producing loci.

### miRNA annotation

Small noncoding RNA pathways contribute to important biological and cellular processes like development, differentiation, and immunity. MicroRNAs (miRNAs) are an endogenous class of small regulatory RNAs that are crucial for post-transcriptional regulation of gene expression [33]. MiRNAs are processed from precursor hairpin structures (pre-miRNAs) which are present in the genome as single-copy loci or, due to gene duplication, as multiple copies of the same miRNA. A comprehensive inventory of *Ae. albopictus* miRNAs is an important resource for investigating small RNA function in vector biology and mosquito antiviral immunity. The official depository of miRNA genes across all species, miRbase [34], does not currently include *Ae. albopictus* miRNAs. Therefore, to annotate miRNA genes in AalbF2, we used the miRDeep2 algorithm [35] on data from small RNA libraries as described above, comprising more than 23 million miRNA-sized 18–24-nt reads. The majority of reads were derived from carcass samples, which is expected as small RNA libraries prepared from ovary samples are more biased towards piRNAs. Initially, miRDeep2 predicted 473 pre-miRNA loci in AalbF2, which was reduced to 229 loci representing 121 distinct pre-miRNA species (Additional file 7) after manual inspection and handling stringent prediction criteria. Among these predictions, 92 represent miRNAs previously annotated in the *Ae. aegypti* genome, three were predicted based on conservation to miRNAs in other insect species, and 26 were entirely novel miRNA genes. Using these predictions, we characterized the expression of miRNAs in ovaries and carcasses and analyzed changes induced by blood feeding. We found that most highly abundant miRNAs show a similar expression pattern between ovaries and carcass (Fig. 3e). Yet, a group of miRNAs, including miR-92a/b, miR-309a, miR-989, miR-2941, miR-2946, and a newly predicted miRNA, miR-new5, were highly abundant (> 1000 reads per million miRNAs; rpmm) exclusively in the ovary samples (Fig. 3e). These findings are coherent with previous studies that identified the clustered miRNAs miR2941/2946 to be specifically expressed in *Ae. aegypti* ovaries [36]. miR-989 is known to be among the most abundant miRNA in mosquito ovaries, both in *Anopheline* and *Aedes* spp. mosquitoes [37, 38]. Similarly, miR-309 was found to be predominantly expressed in *Ae. aegypti* ovary tissue and was furthermore shown to be strongly induced upon blood feeding both in *Aedes* and *Anopheles* spp. mosquitoes [39, 40]. When comparing sugar- and blood-fed *Ae. albopictus*, we observe a similar induction of miR-309a upon blood feeding (Fig. 3f). Likewise, miR-286b and miR-375, which we find to be strongly induced upon blood meal, have previously been shown to be upregulated after blood meal in *Anopheles stephensi* and *Ae. aegypti*, respectively [40, 41], indicating that an orchestrated miRNA response to blood feeding is conserved between different mosquito species. We noted that most newly predicted miRNAs are predominantly expressed in ovary tissue (Fig. 3e), which likely reflects a sampling bias of previous studies that did not deep sequence and predict miRNAs from dissected ovary samples. Some of these predicted miRNA species are relatively highly abundant and are differentially expressed upon blood feeding, suggesting important functions in the physiological processes that are induced upon blood meal.

### Curation of immunity repertoire

The capacity of mosquitoes to acquire, disseminate, and transmit viruses (i.e., vector competence) is a complex phenotype which is controlled by genetic elements of both the vector and the pathogen, as well as environmental variables [42]. Understanding the complex relationship between vectors and pathogens requires understanding innate immunity in mosquitoes. To catalog genes encoding the immune repertoire of *Ae. albopictus*, we searched with BLASTp the predicted peptides of the AalbF2 assembly using as a query 417 manually curated proteins of *Ae. aegypti* from ImmunoDB [43]. We combined phylogenetic comparisons and manual annotation to curate 663 putative immune-related genes encoding 979 predicted proteins, belonging to 27 functional groups (Table 2, Additional file 8). This value is in line with that estimated in AaloF1 (521 genes), confirming the finding that the immune repertoire of *Ae. albopictus* is larger than that of other dipteran species [3, 43]. A manual inspection of the 663 putative immune-related genes using our 5′ and 3′ flanking region pipeline identified a set of 78 suspicious genes that are distributed in half of the immune gene families (Table 2 and Additional file 8), reducing the total number of predicted immune genes to 622.

**Table 2:** The repertoire of immune genes of *Aedes albopictus*. Comparison in the number of immune-related genes among *Ae. albopictus* (Alb, AalbF2, and AaloF1 assemblies), *Ae. aegypti* (Aae, AaegL5), *Anopheles gambiae* (Ag, AgamP4), and *Dr. melanogaster* (Dm, Dmel_r6.29). The numbers in parentheses are the total number of genes after manually excluding suspected artifacts

Gene family or pathway	AalbF2	AaloF1	Aae	Ag	Dm
Antimicrobial peptides (AMPs)	6	11	16	11	7
Autophagy pathway members (APHAGs)	30 (28)	21	20	21	20
Caspase activators (CASPAs)	3	4	4	2	5
Caspases (CASPs)	24 (18)	12	10	15	7
Catalases (CATs)	2	2	2	1	2
CLIP-domain serine proteases (CLIP)	118 (109)	107	82	64	48
C-type lectins (CTLs)	66 (63)	48	44	27	35
Fibrinogen-related proteins (FREPs)	52 (50)	49	38	53	17
Galectins (GALEs)	12	15	12	8	5
Gram-negative binding proteins (GNBPs)	13	13	7	7	3
Heme peroxidases (HPXs)	36 (34)	20	23	23	21
IMD pathway members	16 (13)	11	10	7	8
Inhibitors of apoptosis (IAPs)	5	5	5	7	4
JAK/STAT pathway members (JAKSTATs)	3	4	3	3	3
Lysozymes (LYSs)	7 (6)	9	7	8	11
MD2-like proteins (MLs)	36	26	27	18	10
Peptidoglycan recognition P. (PGRPs)	18 (16)	13	10	7	13
Prophenoloxidase (PPOs)	23 (20)	16	14	9	3
Rel-like NFkappa-B proteins (RELs)	4	4	3	2	3
Scavenger receptor (SCRs)	28 (27)	20	18	16	22
Serine protease inhibitors (SRPNs)	46 (45)	30	26	17	29
Small Reg. RNA pathway Mem. (SRRPs)	51 (49)	41	39	26	24
Superoxide dismutase (SOD)	9	9	6	4	4
Spaetzle (SPZ)	13	13	9	6	6
Thioester (TE)-containing proteins (TEP)	7(5)	3	6	10	6
Toll pathway members (TOLLPATHs)	10	7	6	5	5
Toll receptors (TOLLs)	26 (23)	14	12	9	9
**Total**	**663 (622)**	**527**	**459**	**386**	**344**

Immune system functions can be broadly categorized into three main phases, recognition, signal transduction, and effectors [43–45]. A detailed analysis of the immune repertoire of *Ae. albopictus* revealed extensive expansions in 16 of the 27 functional groups relative to *Ae. aegypti*. In the Toll and IMD pathways, genes involved in recognition and Toll-1/Spz signal transduction show expansion, whereas immune effectors do not display similar family-wide augmentations. Interestingly, while five cecropins (CEC) genes are known in *Ae. aegypti*, we only identified a single CEC gene in the new assembly. We found expansions in families involved in all immune phases of the melanization pathway [46]. The most extreme expansion event regards the CLIP family of regulators with 118 members compared to 67 and 56 genes reported for *Ae. aegypti* and *An. gambiae*, respectively (Table 2). Another interesting case involves the prophenoloxidase (PPO) gene family, which in *Ae. aegypti* includes six tandemly arrayed genes, namely PPO4, PPO8, PPO7, PPO5, PPO1, and PPO2. We found that the entire cluster of six genes has been locally duplicated twice in *Ae. albopictus*, resulting in 18 genes (Fig. 1d, Additional file 2: Table S8). We confirmed this triplication of the clusters using in situ hybridization (Fig. 1b). PPOs are enzymes that catalyze the production of melanin in response to infection [47]. Expansion of PPO genes is not common in insects [48], but in mosquitoes, the number of genes is higher than other insects. The high conservation of the PPO organization and order in the array in both *Ae. aegypti* and *Ae. albopictus* strongly suggests that these duplications are ancient events that occurred 71.4 Mya before the split between the two species [3]. Future studies focusing on dissecting the functional importance of specific family expansions in *Ae. albopictus* may determine their significance for its biology including vector competence and ecological adaptation.

### The sex-determining M locus

In both *Ae. aegypti* and *Ae. albopictus*, sex is determined by a male-determining locus (M locus) that resides on one homolog of chromosome1. *Nix*, the dominant male-determining factor, was first discovered in the M locus of *Ae. aegypti* [49]. We searched AalbF2 for *nix* and located it in an approximately 917 kb scaffold (NW_021838423.1). The *nix* sequence is male-specific as indicated by the chromosome quotient analysis [50] using Illumina reads obtained from male and female mosquitoes of the Foshan strain [11]. A part of the *nix* gene was previously identified in *Ae. albopictus* [49, 51], and its full-length sequence was described in the assembly of the *Ae. albopictus* C6/36 cell line [52]. The *nix* gene in the AalbF2 assembly is annotated as having two exons flanking a small intron (XM_019669557.1), similar to a previous report [5]. However, there is an apparently defective copy of *nix* approximately 22 kb away from XM_019669557.1. This copy does not have an intact open reading frame, and fragments showed up to 70% amino acid identity to XM_019669557.1 (Additional file 1: Fig. S5). Such duplication has not been reported in *Ae. aegypti* [49]. A second gene encoding a myosin heavy chain protein named *myo-sex* [53] has also been shown to be located in the M locus, together with *nix* in *Ae. Aegypti* [5]. Myo-sex is required for male flight in *Ae. aegypti* [54]. A *myo-sex* homolog (XM_019707039.1 or XP_019562584.1; Additional file 1: Fig. S5) has been found in two separate contigs (NW_021838603.1 and NW_021838542.1). It is not yet clear whether the gene that encodes XP_019562584.1 is also located in the M locus in *Ae. albopictus*, as the chromosome quotient analysis [50] was complicated by the presence of highly similar autosomal paralogs (e.g., AALF000603 and XP_019560880).

### Genome-wide polymorphism and linkage disequilibrium

**Fig. 4:**
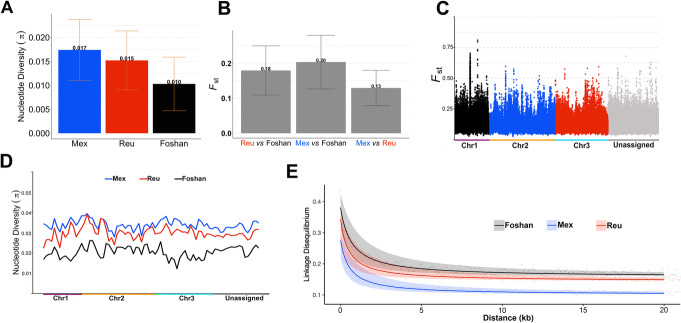
Genome-wide polymorphism in *Aedes albopictus.* **a** Mean nucleotide diversity. **b** Global *F*_ST_, the mean, and standard deviation were calculated from sliding windows analysis. **c** *F*_ST_ estimates between the two wild populations, Tapachula (green) and Tampon (Blue). *F*_ST_ estimates measured across the whole genome with sliding windows of 50 kb with 10-kb steps. Scaffolds that have been assigned to chromosomes 1, 2, and 3 are on the left side of the plot. The remaining unassigned scaffolds are shown on the right side. The unassigned scaffolds were placed in alphabetical order from left to right. **d** Overview of the pattern of nucleotide diversity across the genome. Nucleotide diversity was measured using 50-kb-long sliding windows and 10-kb steps. **e** Linkage disequilibrium (*r*^2^) for two wild populations and a laboratory strain. Red, green, and blue lines represent the fitting curve estimated with the *ngsLD* package, and shaded areas around the lines represent confidence intervals from 100 bootstraps

The level of genetic variability among populations of a given species is the substrate for evolution, which, for an invasive vector species like *Ae. albopictus*, includes processes of adaptation to new ecological settings, selection of resistance alleles against control tools (i.e., insecticides), and co-evolution with pathogens [55–57]. These are biological features important to estimate the epidemiological relevance of *Ae. albopictus* populations and to account for in the design of novel genetic-based strategies of vector control [42, 58]. As for the analyses of the landscape of viral integrations, we used whole-genome sequencing (WGS) data of mosquitoes from Tapachula and Tampon [12] to show the usefulness of AalbF2 in understanding the genomic diversity of *Ae. albopictus* populations. The genetic diversity (*π*) estimates for the laboratory strain are lower than those for the wild populations, which is consistent with the hypothesis of a population bottleneck in the laboratory strain (Fig. 4a). Genetic diversity is slightly higher for the invasive Mexican population than the old population from La Reunion. Global estimates of genetic differentiation (*F*_ST_) among the three samples range from 0.13 to 0.21, with Foshan being the most differentiated (Fig. 4b). Sliding window analyses across the genome showed regions of high and low genetic differentiation between the two wild populations (Fig. 4c) and varying levels of genetic diversity for the two wild populations and the Foshan strain (Fig. 4d). We also derived estimates of linkage disequilibrium (LD). Across the three samples studied, the *r*^2^ Max/2 is approximately 1.3 kb (Fig. 4e). These estimates are strikingly smaller than the estimated values for *Ae. aegypti*, which range between 34 and 101 kb [5]. While comparing these LD, estimates may be complicated by differences in data collection platforms (WGS for *Ae. albopictus* and SNP-chip for *Ae. aegypti*), the striking difference may reflect the different colonization histories of *Ae. aegypti* and *Ae. albopictus* populations [22, 59]. *Aedes aegypti* experienced a slow colonization process that started in the seventh century compared to a quick dispersal in the past 50 years for *Ae. albopictus* that resulted in genetic admixture among the invasive populations [23, 24, 60]. The age of mutations can affect LD with younger mutations, giving higher LD values, it is possible that SNP-chip data and WGS data differ in the average age of mutations, as SNPs are estimated across the whole-genome with no or prior analyses with WGS approaches [61, 62]. The improved continuity of AalbF2 improves our ability to understand the spatial context of genetic signals and long-range patterns.

### Developmental transcriptional profile

**Fig. 5:**
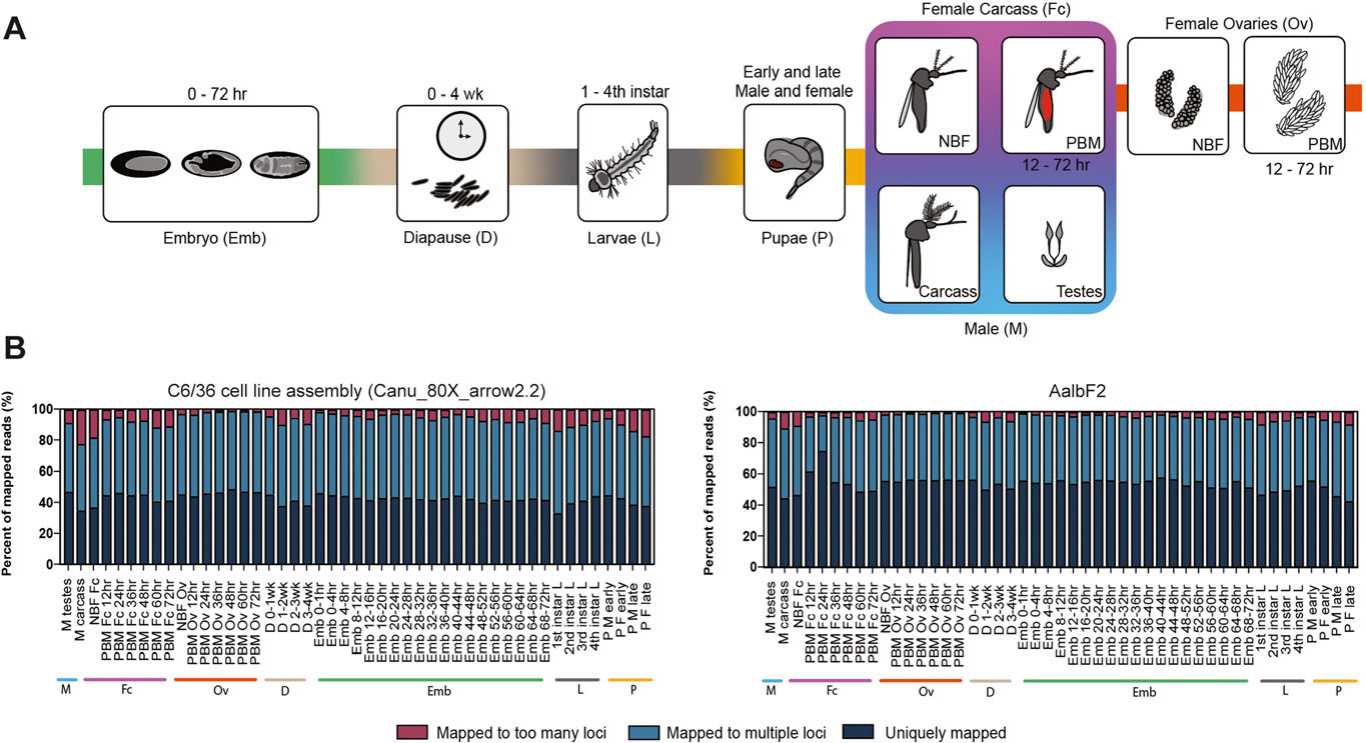
Schematic of mosquito development and mapping statistics of sequenced transcripts to *Ae. albopictus* genome assemblies. **a** Developmental transcriptome of *Ae. albopictus*. Our developmental time course included 34 stages spanning the major developmental groups which are indicated by color bars and are organized as follows: M (blue, male testes, male carcass), Fc (pink, NBF carcass, and multiple time points PBM: 12 h, 24 h, 36 h, 48 h, 60 h, and 72 h), Ov (orange, NBF ovaries, and multiple ovarian timepoints PBM: 12 h, 24 h, 36 h, 48 h, 60 h, and 72 h), D (tan, diapause at multiple time points: 0–1 week, 1–2 weeks, 2–3 weeks, and 3–4 weeks), Emb (embryo at multiple time points: 0–1 h, 0–2 h, 2–4 h, 4–8 h, 8–12 h, 12–16 h, 16–20 h, 20 h–24 h, 24–28 h, 28–32 h, 32–36 h, 36–40 h, 40–44 h, 44–48 h, 48–52 h, 52–56 h, 56–60 h, 60–64 h, 64–68 h, and 68–72 h), L (gray, larvae 1st, 2nd, 3rd, and 4th instar larva stages), and P (yellow, pupae, early male and female, and late male and female pupa stages). **b** Read mapping analysis of *Ae. albopictus* developmental samples against the C6/36 cell line assembly (canu_80X_arrow2.2) and AalbF2 genome assemblies. The distribution reflects the percentage of fragments mapped to too many loci (maroon), fragments mapped to multiple loci (blue), and uniquely mapped fragments (dark blue). There is a significant reduction of duplication in AalbF2 genome assembly compared to the C6/36 cell line (Canu_80X_arrow2.2) genome. More transcripts fell under the uniquely mapped category in the AalbF2 genome

Understanding the network of expression throughout development could provide insights into biological functions implicated in the adaptation of this invasive species to different environments and, coupled with the ability to manipulate genes and their expression, the basis to study gene function. Additionally, *cis*-regulatory elements that guide the expression in a tissue- or time-specific manner could be identified from the analyses of transcriptional profiles and be co-opted in novel genetic-based strategies of vector control. AalbF2 and its predicted gene models served as the basis to establish a comprehensive global view of gene expression dynamics throughout *Ae. albopictus* development taking advantage of recently produced Illumina RNA sequencing (RNA-seq) data from 47 unique samples representing 34 distinct stages of mosquito development [63] (Fig. 5a). These RNA-seq data amounted to 1.56 billion reads corresponding to total sequence output of 78.19 Gb (Additional file 9). A total of 94.1% of the reads were mapped to AalbF2. The number of spliced alignments increased substantially from 39,991,260 in the assembly of the C6/36 *Ae. albopictus* cell line (canu_80X_arrow2.2, *17*) to 56,243,825 in AalbF2 (40.64% increase), again confirming a more complete annotation in AalbF2 (Fig. 5b). The number of uniquely mapped reads also increased significantly likely due to the removal of extensively duplicated regions found in the C6/36 assembly [52].

The analyses of gene expression profiles across all developmental time points showed that the number of expressed genes (transcripts per million ≥ 1) gradually increases through embryogenesis, reaching its highest peak at 68–72 h (Additional file 10). As previously observed, there is an increase in the number of expressed genes during the early pupal stages, and the male germline expresses the highest number of genes among all samples [63]. After a blood meal, female mosquitoes undergo a series of physiological changes to support oogenesis. In PBM ovaries, the number of genes expressed in the female germline changes dramatically from 12 to 36 h.

Pairwise correlation analysis revealed that almost every developmental stage is most highly correlated with its adjacent stage and is very similar to what was previously found (Additional file 1: Fig. S6) [63]. To visualize the various patterns of gene expression and the relationships between the samples, hierarchical clustering and principal component analyses were performed (Additional file 1: Fig. S6).

Based on these analyses, embryos, PBM ovary, pupa, larva, and PBM female carcass samples tend to cluster closer together which is expected since their gene expression profiles are similar as these are developmentally related samples. Two notable exceptions include the male testes and early embryos (0–1 h, 0–4 h, and 4–8 h), likely due to transcripts related to the maternal-to-zygotic transition (Additional file 1: Fig. S6). The male testes sample clusters away from all other samples, reflecting a distinguishing difference between this sample and other samples sequenced (Additional file 1: Fig. S6).

## Discussion

AalbF2 and its associated gene set, databases of nrEVEs, miRNAs, and piRNA clusters are collective resources that will enable great advances in *Ae. albopictus* biology. Additionally, we developed the first physical map of *Ae. albopictus*, which consists of fifty DNA markers that cover the largest genomic scaffolds, rDNA, PPO gene clusters, and the largest viral integration in the genome. Overall, FISH data were consistent with the assembled genome, confirming its large-scale structural accuracy*.* Combining in situ and bioinformatic approaches, we anchored to the *Ae. albopictus* chromosome 58 scaffolds, whose length sum makes 75% of the genome. Analyses of mitotic chromosomes also showed that the *Ae. albopictus* chromosomes are slightly longer than *Ae. aegypti* ones, which is consistent with cytofluorimetry results.

Small RNA analyses identified 121 miRNAs including 26 novel miRNAs, some of which are strongly induced upon blood feeding, suggesting important functions for these miRNAs in reproduction and development. piRNA cluster annotation has provided a high confidence set of piRNA clusters, setting the stage for their inactivation or modification to understand their functions and to explore avenues to exploit them to prevent arbovirus transmission. Moreover, the strong enrichment of newly annotated nrEVE sequences in piRNA clusters provides fuel for the hypothesis that they may provide a potential inherited antiviral defense system [17, 18, 28]. Curation of immunity gene annotation, among the predicted 26,856 protein-coding sequences and the M locus, will unable insights into the immunity pathways that contribute to *Ae. albopictus* vector competence and provide venues for novel genetic-based strategies of control, including those for population suppression based on gene drive systems creating male-biased populations [64]. The developmental transcriptome analysis described here demonstrates that the new genome assembly has produced a significantly more complete gene set with less gene duplications as compared to the previously available genome. The quantification data across developmental time points and multiple tissues will provide the community with an invaluable resource for further exploration of *Ae. albopictus* biology.

## Methods

### Mosquito samples and DNA preparation

*Aedes albopictus* mosquitoes of the Foshan strain are reared at the insectary of the University of Pavia as previously described [11]. We performed a single pair cross between a male and a female individual; from the progeny of this cross, we randomly picked a male and a female and made them mate. We repeated this procedure for six generations, after which we let the progeny of a single-pair mating interbreed. We used pupae from within the 2nd to 3rd generation of the inbred single pair for high-molecular weight (HMW) DNA extraction.

We also used DNA from two wild populations, one from the West African island of La Reunion and Le Tampon city, and one from North America, Mexico, and Jardin Pantheon city. Whole genomic DNA from individual adult mosquito samples was extracted using the QIAGEN Blood and Tissue kit (Qiagen, Hilden, Germany) following the manufacturer’s instructions. PCR-free sequencing libraries were prepared using a custom pipeline including the TruSeq DNA PCR-Free kit (Illumina) [65]. Samples were sequenced on the Illumina HiSeqX sequencing platform pooling 32 samples per flowcell at Verily Life Sciences (South San Francisco, CA) resulting in an average of 100 million 150-bp reads per sample as previously described [12].

### Flow cytometry

The genome size of *Ae. albopictus* mosquitoes from different strains was estimated by flow cytometry as previously described [66]. Briefly, the nuclei were released from the heads of a mosquito and a *Drosophila virilis* standard (1C—328 Mb) in 1 ml of cold Galbraith buffer using 15 strokes of a pestle in a 2-ml Kontes Dounce Tissue grinder. The released nuclei were filtered through 40-μm nylon mesh, stained with 25 ml of 1 mg/ml propidium iodide, allowed to stain for 3 h in the cold and dark, and then scored for relative red (PI) fluorescence using a Cytoflex flow cytometer. The 1C genome size of the sample was estimated as the ratio of the relative fluorescence of the 2C peaks of the sample and standard multiplied times the 1C amount of DNA in the standard. A minimum of 1000 nuclei were scored under each peak. All scored peaks were symmetric with a CV below 2.0.

### Pacific Biosciences library construction and sequencing

HMW DNA extraction for Pacific Bioscience sequences by the Berkley genome facility which also built and sequenced libraries. To obtain HMW DNA, fresh frozen pupae (around 80 male sibling pupae) were disassembled using a Pyrex mortar in 2-ml ATL with 4 μl RNase. Samples were then incubated at 37 °C for 30 min with a parafilm cover and gentle agitation (300 rpm). After the addition of 100–200 μl proteinase K, samples were incubated overnight at 37 °C overnight. DNA was then purified from proteins using a standard phenol-chloroform extraction protocol, followed by precipitation in 100% ice-cold ethanol. DNA was then washed with 70% ethanol at room temperature (max speed spins 10 min). Purified DNA was resuspended in elution buffer with no EDTA, and samples were left rotating slowly overnight at 4 °C to resuspend.

### Contig assembly and polishing

Initial contigs were generated using Canu v1.7.1 with the parameters:

‘genomeSize=2g’ ‘correctedErrorRate=0.105’ ‘corMinCoverage=4’ ‘corOutCoverage=all’ ‘corMhapSensitivity=normal’ ‘gridOptions=--time=72:00:00 --partition=norm’ ‘stageDirectory=/lscratch/$SLURM_JOBID’ ‘gridEngineStageOption=--gres=lscratch:100’ ‘ovlMerThreshold=500’.

Following assembly, consensus polishing was run on all contigs using the Arrow software included with PacBio SMRTAnalysis 5.1.0.26412 (https://www.pacb.com/products-and-services/analytical-software/smrt-analysis/), using Minimap2 v2.11 [67] and pbbamify for the read mapping stage. Due to the large assembly size observed, secondary alleles were identified and removed using two approaches. First, Purge Haplotigs (commit: 6414f68101103af33d47650ea84623a26343bda1) was run with the following commands:

minimap2 -ax map-pb --secondary=no -t 16 asm.contigs.fasta reads.fasta.gz > reads.sam

samtools view -b -T asm.contigs.fasta -S reads.sam > reads.bam

samtools sort -O bam -o reads.sorted.bam -T tmp reads.bam

samtools index reads.sorted.bam

purge_haplotigs readhist reads.sorted.bam

purge_haplotigs contigcov -i reads.sorted.bam.genecov -l 5 -m 33 -h 55 -j 200

purge_haplotigs purge -t 32 -g asm.contigs.fasta -c coverage_stats.csv -b reads.sorted.bam -windowmasker

Following Purge Haplotigs, an additional custom program was run to identify BUSCO genes present on multiple contigs (https://github.com/skingan/HomolContigsByAnnotation). All contigs flagged by either Purge Haplotigs or the BUSCO analysis were considered secondary alleles and removed from the primary assembly. The primary assembly contigs were then scaffolded using SALSA v2.0 with the command:

run_pipeline.py -a asm.fasta -l asm.fasta.fai -b alignment.filtered.bed -o scaffolds_new -c 10000 -i 20 -e GATC -m yes

The scaffolded primary contigs are considered the AalbF2 assembly, and the alternative alleles were left as unscaffolded contigs but included in the submitted assembly for completeness.

All BUSCO results were generated using BUSCO v3 [8] using the “diptera_odb9” ortholog database. The numbers of rRNA genes in the genome assemblies was estimated using barrnap (https://github.com/tseemann/barrnap) with HMMER 3.1 and “eukaryotes” setting.

### Comparative alignment of DNA-seq and RNA-seq data to AalbF2 and AaloF1

Whole-genome sequencing (WGS) and total RNA sequencing data obtained from uninfected *Ae. albopictus* Foshan mosquitoes were downloaded from NCBI Sequence Reads Archive (SRA) [11], BioProject PRJNA475859. Reads quality was assessed with FastQC (https://www.bioinformatics.babraham.ac.uk/projects/fastqc/). WGS and RNA-seq reads were separately aligned to AalbF2 and AaloF1. WGS and RNA-seq reads were aligned using MAGIC-Blast [68] and HISAT2 [69] with default parameters, respectively. Alignments were sorted with BamTools Sort, and alignment statistics were calculated with BamTools Stats [70].

### In situ hybridization and physical map construction

We developed a new mapping approach based on the amplification of DNA probes using cDNA instead of bacterial artificial chromosome (BAC) clones. DNA probes derived from the largest genomic scaffolds, 18S rDNA, PPO genes, and Canu-Flavi19 were mapped to the chromosomes using FISH. To identify genes that could be used for the physical mapping of the *Ae. albopictus* genome, transcripts of *Ae. albopictus* C6/36 cell lines were aligned against AalbF2 [52]. DNA fragments were amplified by PCR using a Q5 high-fidelity DNA polymerase (New England Biolab, Ipswich, MA, USA). cDNA or genomic DNA fragments were used as templates to amplify transcript fragments or large exons; cDNA or genomic DNA fragments were used as templates to amplify transcript fragments or large exons, respectively. RNA was obtained from mosquito ovaries following the Zymo Research Direct-Zol DNA/RNA mini prep protocol (Zymo Research Corporation, Irvine, CA, USA). cDNA was synthesized using ~ 200 ng RNA and primed with oligo (dT) following the Thermo Fisher Scientific Superscript III first-stand synthesis system protocol (Thermo Fisher, Ashville, NC, USA). Laboratory protocols for performing preparations and principal steps for FISH have been described earlier [71, 72]. Transcript fragments or large exons with minimal length of 3.8 kb were used as probes for FISH. PCR-amplified DNA was labeled with two fluorescence dyes Cy3- or Cy5-dUTP (Enzo Life Sciences, Farmingdale, NY, USA) by nick-translation. A pair of DNA probes was hybridized simultaneously to the chromosomes [71, 73]. Slides of mitotic chromosomes were prepared from imaginal discs of 4th instar larvae from the Foshan strain following the published protocols [71, 72, 74]. Chromosomes were stained with a YOYO-1 dye (Thermo Fisher, Ashville, NC, USA), and slides were mounted with a Prolog Gold reagent (Thermo Fisher, Ashville, NC, USA). FISH results were analyzed using a Zeiss LSM 880 Laser Scanning Microscope (Carl Zeiss Microscopy, LLC, White Plains, NY, USA) at × 600 magnification. Chromosome idiograms were developed using previously described protocols [72, 74]. Chromosome proportions, such as relative chromosome length and centromeric index (relative length of the p arm), were calculated based on measurements of 60 chromosomes. The statistical analysis was performed using the JPM Pro 15 software program at 95% confidence intervals [75]. One-way ANOVA was used to calculate *P* values for comparison chromosome proportions between *Ae. albopictus* and *Ae. aegypti*. Chromosomes were subdivided into 96 bands with 4 different intensities.

### Pair-wise comparison between *Aedes aegypti* chromosomes and *Aedes albopictus* scaffolds

The *Ae. aegypti* AaegL5 genome assembly was downloaded from VectorBase (https://www.vectorbase.org/). The first 58 scaffolds of AalbF2 (corresponding to the L75 of the assembly) were aligned to *Ae. aegypti* chromosomes with minimap2 [67]. Only hits with a percentage of identity higher than 40% were retained. Alignment results were summarized and visualized as a comparative genome dot plot using D-GENIES (http://dgenies.toulouse.inra.fr/).

### Identification of *Aedes albopictus* nrEVE

The AalbF2 genome assembly was screened for integrations from nonretroviral RNA viruses using a blast-based approach [76]. To this purpose, a database of viral proteins was created. The database included all complete amino acid sequences belonging to ssRNA, dsRNA, and unclassified RNA viruses with a tropism for vertebrates present in the NCBI RefSeq database as of August 2018 (Additional file 4). The database was updated including the *Xinmoviridae* and *Phenuiviridae* families. Candidate viral integrations were identified using the AalbF2 genome assembly as a query and the viral database and running the BLASTx [76] algorithm with an *e* value threshold of 1e^−6^. Resulting hits were merged and refined with the EveFinder Pipeline [17]. Putative viral integrations were blasted against all proteins available in the NCBI RefSeq and non-redundant (NR) database, and a custom pipeline was used to recognize and remove false positive, including sequences with certain homology to eukaryotic proteins. Additionally, viral integrations closer than 100 bp and derived from the same viral species were joined. Each viral integration was assigned to a viral family based on its most similar virus in the NR database. The upstream and downstream 1-kb regions of each viral integration were inspected for repeated elements using a custom script based on BLASTn and a database of *Ae. albopictus* repeats predicted using RepeatModeler with default settings (http://www.repeatmasker.org/RepeatModeler/). This database was used to run RepeatMasker (http://www.repeatmasker.org) with default parameters to find and classify TEs (Additional file 2: Table S2).

Whole-genome sequencing (WGS) data of mosquitoes from La Reunion and Mexico were analyzed with VyPER [20], followed by custom scripts, to verify for the presence of additional viral integrations, different than what was characterized in AalbF2. Bioinformatic predictions of each novel viral integration were molecularly tested by PCR using specific primers (Additional file 2: Table S7).

The correspondence between AaloF1 and AalboF2 viral integrations was analyzed using a BLASTn-based script (Additional file 2: Table S5). Viral integrations annotated in AalbF2 were also used to test whether the haplotig purging pipeline effectively moved to the secondary assembly alternative haplotypes. BLASTn was used to find hits in the secondary assembly for each viral integration with the exception of the unclassified and *Chuviridae*-like nrEVEs, which are too redundant among themselves to provide reliable results. Hits were retained when at least 98% of the query length was present with a minimum percentage of identity of 95% (Additional file 2: Table S6).

### piRNA cluster annotation

One-week-old female mosquitoes from the Foshan strain were provided with a 2-ml rabbit blood meal at the Institut Pasteur (Paris). A total of 60 fully engorged females were kept at 28 °C by feeding on a 10% sucrose solution ad libitum. Thirty females were collected at 14 and 21 days post-blood meal (PBM). Each female was dissected, the ovaries were removed from carcasses, and both ovaries and carcasses were pooled into two groups of 15 for each time point. In parallel, 60 mosquitoes were kept on a sugar diet under the same conditions and sampled as described before. Total RNA was extracted from each pool using the Nucleospin miRNA kit by Macherey Nagel following the manufacturer’s instructions. Extracted RNA was sent to the Beijing Genomics Institute (BGI) for sequencing. Total RNA was used for custom DNBseq library preparation and sequenced on a BGI-SEQ 500 to obtain 40 million reads SE50 per sample. Small RNA sequencing data were deposited to the SRA (BioProject PRJNA607026).

Ambiguous (multi-mapping) reads from the small RNA-seq libraries described above were either randomly distributed over all possible mapping positions (--best –strata -M1), or alternatively, ambiguous mapping reads were excluded (-m1) to obtain all uniquely mapping reads unambiguously assigned to single piRNA loci. For piRNA cluster annotation, reads in the size range from 25 to 30 bp were normalized to one million mapped piRNAs [ppm] to account for the lower amount of piRNAs relative to other sRNA classes in somatic tissues compared to the germline, and piRNAs were trimmed to their 5′ terminal nucleotide. Clusters were annotated similar to the approach used in fruit flies [31], optimizing minimal requirements for a larger and more repetitive genome like *Aedes albopictus* (Additional file 1: Fig. S4). Briefly, the genome was scanned with non-overlapping 5-kb windows; windows with 10 or more ppm and a maximum distance of 5 kb were merged into a cluster. Clusters were then filtered for being covered by at least 5 unique ppm, mapping to at least 5 different positions. Borders of the clusters were defined by the two furthest piRNAs, and clusters that were either very small (< 1 kb) or large but only covered by few piRNAs (piRNA density < 10 ppm/kb) were excluded. We performed separate annotations for germline and soma to avoid averaging out clusters that are only expressed in one but not the other tissue and that might fall below some of the set thresholds. The final dataset of piRNA clusters was obtained by merging the two datasets, and two clusters that were exclusively determined by rRNA reads were manually excluded from the list. piRNA cluster annotation was solely guided by piRNA coverage of the respective genomic regions but did not include assumptions on nucleotide biases or strand asymmetry, as used for example for the annotation of piRNA clusters in *Aedes aegypti* Aag2 cells [17] as culicine mosquitoes encode developmentally relevant piRNAs without 1 U bias [77].

The expression of clusters was confirmed and quantified using small RNA libraries not used for the initial cluster annotation. Expression was normalized to one million mapped piRNAs to compare somatic and germline tissues with different proportions of piRNAs among total small RNAs, or to million mapped small RNAs to plot coverage of the clusters. Enrichment of repeat classes was calculated as the quotient of the genomic fraction of nucleotides annotated with the respective repeat in clusters compared to the whole genome.

### miRNA predictions and expression analysis

Small RNA libraries from samples collected 14 days PBM were mapped to the new AalbF2 assembly or, alternatively, the previous AaloF1 assembly with bowtie (v1.2.2) [78] without allowing mismatches. Mapped small RNA reads were size selected for 18–24 nucleotides, converted into a single concatenated fasta file comprising 23,644,778 reads and used as input for the mapping module of miRDeep2 [35]. The program (Galaxy version 2.0.0.8.) was accessed through the Mississippi Galaxy instance available at http://mississippi.fr and the settings were –k 19 –m –p –r 100. The obtained output files in fasta and ARF format were used as input for the miRDeep2 module together with a list of known precursor and mature miRNA sequences from the *Ae. aegypti* genome, downloaded in fasta format from miRBase 28 May 2019 [34]. In addition, precursor miRNAs from *Culex quinquefasciatus*, *Anopheles gambiae*, *Drosophila melanogaster*, *Apis mellifera*, and *Bombyx mori* were used as input. All other settings were left as default, and a detailed fasta output was requested. The resulting tabular output file was split into three lists: (1) known and predicted miRNAs based on the *Ae. aegypti* reference datasets, (2) known but unpredicted miRNAs, and (3) novel predicted miRNAs which include predictions supported by the reference data from other insect species provided as well as entirely new predictions. The list of known miRNAs was inspected for miRNA predictions in which the known 3′ miRNA was mapped on a 5′ arm of a putative hairpin and vice versa. These isoforms generally had very low miRDeep scores compared to the true copy (3p miRNA mapped on 3′ arm and/or 5p miRNA mapped on 5′ arm) and were manually deleted from the list. From the list of known but unpredicted miRNA, only predicted miRNAs that were supported by at least 10 mature miRNA counts were considered. Their genomic position is not provided in the miRDeep2 program and was determined using the NCBI BLASTn algorithm with the pre-miRNA sequences from the *Ae. aegypti* genome and the AalbF2 assembly as query and subject inputs, respectively. The list of novel miRNA predictions was manually curated using the following stringent parameters [79]. More than 80% of the mature miRNAs were required to have the same 5′ end on the precursor. More than 80% of the predicted miRNA star reads were required to start and end at nucleotide positions predicted to give rise to a characteristic Drosha/Dicer product allowing a margin of ± 1 bp at both the 5′ and 3′ ends. Predictions that were not supported by any predicted miRNA star read were excluded unless the precursor showed high similarity to a known insect miRNA and was supported by > 1000 mapped reads. Precursors with > 1000 BLAST hits were also excluded. miRNA expression analysis was performed in the public server of the Galaxy toolshed [80] using small RNA datasets as described above. Small RNAs were mapped to the AalbF2 assembly and their genomic positions were intersected with the location of known and predicted pre-miRNAs obtained from the miRDeep2 analysis using BEDtools intersect intervals (Galaxy Version 2.29.0; settings: *same* strand, -wo,-abam). The obtained output was filtered for an overlap of small RNA reads and miRNA precursors of at least 18 bp and no more than 24 bp. The occurrence of each pre-miRNA was then counted, and raw counts were exported to Microsoft Excel. The read count per pre-miRNA was normalized to the total number of miRNA reads in each dataset and expressed as reads per million miRNAs (RPMM). Where indicated counts were transformed to log_2_(RPMM + 1). Expression data were plotted in GraphPad Prism.

### Generation of RefSeq gene set annotation

The NCBI Eukaryotic Genome Annotation Pipeline was used to annotate genes, transcripts, and proteins on the primary assembly of AalbF2, Aalbo_primary.1 (accession GCF_006496715.1). Due to the highly repetitive nature of the genome, masking was done with RepeatMasker using a collection of repeats generated with RepeatModeler [52] and WindowMasker [81] and resulted in 74% of the genome being masked. Nearly 8 billion RNA-seq reads from 170 *Ae. albopictus* BioSamples were retrieved from SRA and aligned to the masked genome using BLAST [82] followed by Splign [83], along with 366 known RefSeq transcripts, 6046 GenBank transcripts, and 302,415 ESTs from the *Aedes* genus. The set of proteins aligned to the masked genome consisted of 30,044 known RefSeq proteins from *Dr. melanogaster*, 27,814 model RefSeq proteins from *Ae. aegypti*; 100,517 GenBank proteins from insects, 1084 known RefSeq proteins from *Nasonia vitripennis*, and 528 known RefSeq proteins from *Apis mellifera*. The gene models’ structures and boundaries were primarily derived from these alignments. Ab initio extension and joining/filling of partial ORFs in compatible frame were performed by Gnomon (https://www.ncbi.nlm.nih.gov/genome/annotation_euk/gnomon/), using a hidden Markov model trained on *Ae. albopictus* where alignments did not define a complete model but the coding propensity of the region was sufficiently high to predict a coding gene with confidence. tRNAs were predicted with tRNAscan-SE:1.23 [84], and small non-coding RNAs were predicted by searching the RFAM 12.0 HMMs for eukaryotes using cmsearch from the Infernal package [85]. The annotation of the Aalbo_primary.1 assembly, *Ae. albopictus* Annotation Release 102 (https://www.ncbi.nlm.nih.gov/genome/annotation_euk/Aedes_albopictus/102/) or AR 102 resulted in 26,856 protein-coding genes (84% fully supported by experimental evidence, and 12% with more than 5% ab initio), 9530 non-coding, genes and 4108 pseudogenes.

### Artifacts and gene duplication detection in AalbF2

To identify highly similar sequences in AalbF2 while obtaining their position in the scaffolds, we performed a BLASTp all_vs_all of the 40,086 peptide sequences with an *e* value of 1e^−40^. After excluding self-alignments, we extracted the sequences of suspected gene duplications including the 500 bp and 1000 bp in both the 5′ and 3′ flanking regions of the coding sequence. A BLASTn analysis using as queries the 500-bp gene regions and 1000-bp gene dataset against the new assembly was then performed (Additional file 1: Fig.S1, step 3). All matches with 100% coverage over the entire sequence, and 98% of identity were filtered and collated into candidate artifact pairs list.

### Identification of immunity genes and manual curation of their annotation

A protein sequence homology analysis pipeline was developed to identify immune-related genes in AalbF2. A dataset of 417 manually curated protein sequences from 27 immune functions of *Ae. aegypti* [8] was used as a query to search by BLASTp against the peptide database (GCF_006496715.1). Local alignments were selected based on the associated *e* value 1e^−20^ and a cutoff of ≧ 60% of identity. This was followed by sequence extraction and filtering of isoform sequences and comparative analyses and manual curation to map synteny, phylogeny, and sequence identity. Gene duplication events were detected using as a reference the orthologous immune-related genes of *Ae. aegypti*. We also performed a genome mapping analysis to uncover paralogous generated by tandem duplications. The evolutionary history of each expanded immune family protein was then inferred using a maximum Likelihood method with Phylogeny.fr platform [86]. The pipeline One-Click mode setting was used as default, which includes MUSCLE for multiple alignments [87], Gblocks for alignment curation [88]. Improvement of phylogenies was done after removing divergent and ambiguously aligned blocks from protein sequence alignments [89], and TreeDyn for tree drawing was used to reconstruct a robust phylogenetic tree from a set of sequences [90]. The nwk files obtained were edited in the iTOL platform (https://itol.embl.de/login.cgi) and exported as SVG files.

Orthogroups, orthologues, and single-copy gene clusters of immune-related genes across multiple species were defined by clustering the immune-related peptides of *Ae. albopictus* and the complete peptides of *Ae. aegypti* (Liverpool-AaegL5 assembly), *An. gambiae* (PEST-AgamP4), and *Dr. melanogaster* (Dmel-r6.26) species. Two approaches were performed using OrthoVenn2 [91] and OrthoFinder [92]. Parameters for OrthoVenn2 considered the *e* value cutoff for all-to-all protein similarity comparisons, the inflation value for the generation of orthologous clusters using the Markov cluster algorithm (*e* value = 1 × 10^−2^ and inflation value = 1.5).

### Analyses of the sex-determining M locus

The annotated *Ae. albopictus nix* transcript (XM_019669557 or LOC109397226) was used as a query to perform a BLASTn (*e* value cutoff 1e^−5^) against *AalbF2*. A 1436-bp mRNA sequence showed a 100% match to contig NW_021838423.1 from position 209,080 to 210,622, except for a small intron in the genomic sequence. When the *Ae. albopictus* NIX protein sequence (XP_019525102) was used as a query to perform tBLASTn against AalbF2, a possibly duplicated copy was found (Additional file 1: Fig. S5) in addition to the annotated LOC109397226. Although the precise beginning of the open reading frame of the duplicated copy is unclear, the duplicated copy is likely to be approximately 20 kb away from the annotated nix gene. It is not clear whether the duplicated copy is functional as its open reading frame appears to be interrupted by premature stop codons and indels (Additional file 1: Fig. S5). The duplicated copy is significantly related to and only to NIX at the amino acid level. The duplication appears to have occurred a long time ago as the previously mentioned BLASTn searches did not show a significant match between the annotated *Ae. albopictus* nix transcript (XM_019669557 or LOC109397226) and the duplicated copy (*e* value cutoff 1e^−5^). Male specificity of both LOC109397226 and the duplicated nix sequences was confirmed by using the chromosome quotient analysis [50] with Illumina reads obtained from Foshan strain male and female mosquitoes [11]. The *Ae. aegypti* myo-sex protein sequence [53] was used in a tBLASTn search to identify the *A. albopictus* homologs of myo-sex, and phylogenetic analysis was conducted using Phylogeny.fr [86, 93].

### Analyses of genome-wide polymorphism and linkage disequilibrium

We processed WGS datasets of mosquitoes from the Foshan strain, La Reunion, and Mexico [11, 12] to discover single nucleotide polymorphism (SNP) and derive estimates of linkage disequilibrium (LD) and other population genetics parameters. Paired-end reads were aligned to AalbF2 using BWA-MEM version 0.7.17 [94]. We discarded unmapped reads as well as reads with mapping quality below a mapQ of 30 using SAMtools version 1.9 [95]. Next, we used SAMtools to merge and sort the paired- and single-end pseudoreads read alignments into a single BAM file to be used in subsequent analyses. First, we used GATK version 3.8 [96]. to perform realignments around indels. Second, we used Picard tools version 2.9.0 (https://broadinstitute.github.io/picard/) to remove optical and PCR duplicates. Third, we generated an uncompressed BCF using SAMtools mpileup version 1.3.1 with indel calling disabled, skipping bases with baseQ/BAQ less than 30, and with mapQ adjustment (-C) set to 30. Fourth, we converted it to a VCF file using bcftools version 1.5. (http://samtools.github.io/bcftools/bcftools.html) We filtered out low-quality SNPs with SNPcleaner version 2.4.1 [97] and removed sites that had a total depth across all individuals less than 1500 reads or had less than 10 individuals with at least two reads each. Finally, additional sites were filtered out based on the default settings within the SNPcleaner script. We obtained a set of robust sites for each population comprising the sites that passed all our filtering thresholds. We restrict our analyses to these robust sites using the option -sites of ANGSD version 0.929-21 [98]. Within ANGSD, we used uniquely mapped reads with minimum map quality and base quality thresholds of 30 and 20, respectively. For linkage disequilibrium (LD) analyses we used ANGSD genotype likelihoods to directly estimate decay using ngsLD version 1.1.0 [99]. We used ANGSD to calculated global Weir and Cockerham *F*_ST_ [100] between populations and diversity (*π*) within populations directly from the estimated allele frequencies from the sequencing read data. We obtained approximately 359 million robust sites per population during our filtering. We then performed a sliding window analysis to estimate *F*_ST_ and *π* across all scaffolds of the new genome with 50,000-bp windows and 10,000-bp steps, with a total of 85,844 windows. We plotted windows with at least 2000 sites with each window being a point in the plots. We estimated the pairwise LD using the ngsLD package [99], which takes the uncertainty of genotype assignment into account by avoiding hard call genotypes entirely and using genotype likelihoods (GLs). The program has two algorithms to estimate LD levels from GLs. One is a maximum likelihood approach to estimate the haplotype frequencies between pairs of sites to estimate *D*, *D*′, and *r*^2^ and the other is based on the squared Pearson correlation (*r*^2^) between expected genotypes using their posterior probabilities. All LD estimates were done with 100 bootstraps, and we tested different bin sizes until we obtained small confidence intervals. We estimated the LD pairwise comparisons for all sites and randomly picked 0.01% of the comparisons to run the ngsLD algorithms for fitting and plotting. The 0.01% sampling data points represent at least 1.5 million *r*^2^ comparisons. We used new and previously publish SNP chip data from *Ae. aegypti* to estimate LD for this species and compare to our results [5]. We generated our plots in R using the built-in functions and the R packages ggplot2 [101], Sushi [102], and qqman [103].

### Developmental profile analyses

We used wild-type *Ae. albopictus* mosquitoes from San Gabriel Valley, located in the Los Angeles County, CA, for RNA extraction. Mosquito rearing, total RNA isolation, and RNA-seq were carried out as previously described [63]. RNA-seq libraries were aligned to AalbF2 using STAR aligner [104]. Gene models were downloaded from NCBI (GCF_006496715.1_Aalbo_primary.1_genomic.gtf) and quantified with featureCounts [105]. Transcripts per million (TPM) and fragments per kilobase million (FPKM) values were calculated from count data using Perl scripts. All sequencing data has been made publicly available at NCBI SRA under BioProject PRJNA563095 (genomic) and PRJNA563095 (transcriptomic).

## Supplementary information

**Additional file 1:** Document containing supplementary figure S1 to S6.

**Additional file 2:** Document containing supplementary tables S1 to S8.

**Additional file 3:** Metadata including the number of duplication times for 500 bp and 1000 bp flanking regions and the list of the genomic regions with putative artifact duplications.

**Additional file 4:** Database of viral species used for nrEVEs screening in AalbF2.

**Additional file 5: **Database of nrEVEs in the *Aedes albopictus* genome (AalbF2).

**Additional file 6: **Database of miRNAs in the *Aedes albopictus* genome (AalbF2).

**Additional file 7: **Database of piRNA clusters in the *Aedes albopictus* genome (AalbF2).

**Additional file 8:** Immunity genes annotated in AalbF2.

**Additional file 9:** Metadata of sequenced dataset and read mapping analysis of Ae. albopictus transcripts.

**Additional file 10:** Number of genes with counts>0 and TPM≥1 and Gene expression in transcripts per million. (TPM) for RNA-Seq libraries from developmental timepoints.

**Additional file 11:** Gene expression in counts for RNA-Seq libraries from developmental timepoints. Gene counts were extracted with featureCounts.

**Additional file 12:** Review history.

## Data Availability

All the data that support the genome assembly described in this study have been deposited in the NCBI repository and can be accessed with the BioProject accession ID PRJNA530512 [106]. Alternative haplotypes discarded from the primary assembly can be accessed with the BioProject accession ID PRJNA535494 (https://www.ncbi.nlm.nih.gov/bioproject/535494). The publicly available *Ae. albopictus* data analyzed during the current study are available from the NCBI BioProject repository under the following accession IDs: PRJNA484104 (https://www.ncbi.nlm.nih.gov/bioproject/484104) for the Foshan strain WGS data [11]; PRJNA484104 (https://www.ncbi.nlm.nih.gov/bioproject/PRJNA484104) and PRJNA562979 (https://www.ncbi.nlm.nih.gov/bioproject/PRJNA562979) for the wild samples WGS data [12]; PRJNA475859 (https://www.ncbi.nlm.nih.gov/bioproject/PRJNA475859) for the Foshan RNA-seq data [13]; PRJNA563095 (https://www.ncbi.nlm.nih.gov/bioproject/PRJNA563095) for the RNA-seq data pertaining to the developmental transcriptome profiles [63]; PRJNA607026 (https://www.ncbi.nlm.nih.gov/bioproject/PRJNA607026) for the small RNA-seq data used to analyze piRNA and miRNA expressed by *Ae. albopictus* [12]. The scripts and pipeline for the identification of novel viral integrations described in this study are available on GitHub (https://github.com/epischedda/ViR).
