# Author Correction: Improved reference genome of the arboviral vector *Aedes albopictus*

**DOI:** 10.1186/s13059-021-02431-x

**Published:** 2021-07-12

**Authors:** Umberto Palatini, Reem A. Masri, Luciano V. Cosme, Sergey Koren, Françoise Thibaud-Nissen, James K. Biedler, Flavia Krsticevic, J. Spencer Johnston, Rebecca Halbach, Jacob E. Crawford, Igor Antoshechkin, Anna-Bella Failloux, Elisa Pischedda, Michele Marconcini, Jay Ghurye, Arang Rhie, Atashi Sharma, Dmitry A. Karagodin, Jeremy Jenrette, Stephanie Gamez, Pascal Miesen, Patrick Masterson, Adalgisa Caccone, Maria V. Sharakhova, Zhijian Tu, Philippos A. Papathanos, Ronald P. Van Rij, Omar S. Akbari, Jeffrey Powell, Adam M. Phillippy, Mariangela Bonizzoni

**Affiliations:** 1 Department of Biology and Biotechnology, University of Pavia, 27100 Pavia, Italy; 2 Department of Entomology and the Fralin Life Science Institute, Virginia Polytechnic and State University, Blacksburg, VA 24061 USA; 3 Department of Ecology and Evolutionary Biology, Yale University, New Haven, CT 06511-8934 USA; 4 Genome Informatics Section, Computational and Statistical Genomics Branch, National Human Genome Research Institute, National Institutes of Health, Bethesda, MD 20892-2152 USA; 5 National Center for Biotechnology Information, National Library of Medicine, National Institutes of Health, Bethesda, MD 20894 USA; 6 Department of Entomology, Robert H Smith Faculty of Agriculture, Food and Environment, The Hebrew University of Jerusalem, 7610001 Rehovot, Israel; 7 Department of Entomology, Texas A&M University, College Station, TX 77843 USA; 8 Department of Medical Microbiology, Radboud University Medical Center, Radboud Institute for Molecular Life Sciences, P.O. Box 9101, 6500 HB Nijmegen, The Netherlands; 9 Verily Life Sciences, South San Francisco, CA 94080 USA; 10 Division of Biology and Biological Engineering, California Institute of Technology, Pasadena, CA 91125 USA; 11 Department of Virology, Arbovirus and Insect Vectors Units, Institut Pasteur, 75015 Paris, France; 12 Laboratory of Evolutionary Genomics of Insects, The Federal Research Center Institute of Cytology and Genetics, Siberian Branch of the Russian Academy of Sciences, Novosibirsk, 630090 Russia; 13 Laboratory of Ecology, Genetics and Environment Protection, Tomsk State University, Tomsk, 634041 Russia; 14 Division of Biological Sciences, University of California, San Diego, La Jolla, CA 92093-0349 USA

**Correction to: Genome Biol 21, 215 (2020)**

**https://doi.org/10.1186/s13059-020-02141-w**

Following publication of the original article [1], the authors identified an error in the section "Distribution and structure of piRNA clusters" on page 7. The value of the SD is not correct. Instead of a SD 634.885 Kb, it should be SD 16.79.

The correct statement should read: “We used small RNA libraries generated from somatic tissues (female carcass) as well as germline tissues (ovaries) to annotate 1441 piRNA clusters with an average size of 10.911 kb (SD 16.79 kb; max: 139.92 kb) … ”
