# Supplementary material for: Improved reference genome of the arboviral vector Aedes albopictus
Source: Genome Biol. 2020 Aug 26;21:215. doi: 10.1186/s13059-020-02141-w (PMC7448346; doi:10.1186/s13059-020-02141-w)
Supplement: Supplementary file 1 — Additional file 1: Document containing supplementary figure S1 to S6. [file 13059_2020_2141_MOESM1_ESM.docx]

Additional File 1 for

Improved reference genome of the arboviral vector *Aedes albopictus*

Umberto Palatini, Reem A. Masri, Luciano V. Cosme, Sergey Koren, Françoise Thibaud-Nissen, James K. Biedler, Flavia Krsticevic, J. Spencer Johnston, Rebecca Halbach, Jacob E. Crawford, Igor Antoshechkin, Anna-Bella Failloux, Elisa Pischedda, Michele Marconcini, Jay Ghurye, Arang Rhie, Atashi Sharma, Dmitry A. Karagodin, Jeremy Jenrette, Stephanie Gamez, Pascal Miesen, Patrick Masterson, Adalgisa Caccone, Maria V. Sharakhova, Zhijian Tu, Philippos A. Papathanos, Ronald P. Van Rij, Omar S. Akbari, Jeffrey Powell, Adam M. Phillippy, Mariangela Bonizzoni

Correspondence to: [m.bonizzoni@unipv.it](mailto:m.bonizzoni@unipv.it)

**Additional file 1 includes supplementary figures S1 to S6**

**Contents:**

Fig. S1: Solving excess heterozygosity

Fig. S2: Correspondence between *Ae. aegypti* and *Ae. albopictus*.

Fig. S3: Viral integrations of the *Ae. albopictus* genome.

Fig. S4: piRNA cluster annotation.

Fig. S5: Insights into the M locus of *Ae. albopictus*.

Fig. S6: Global dynamics of gene expression.


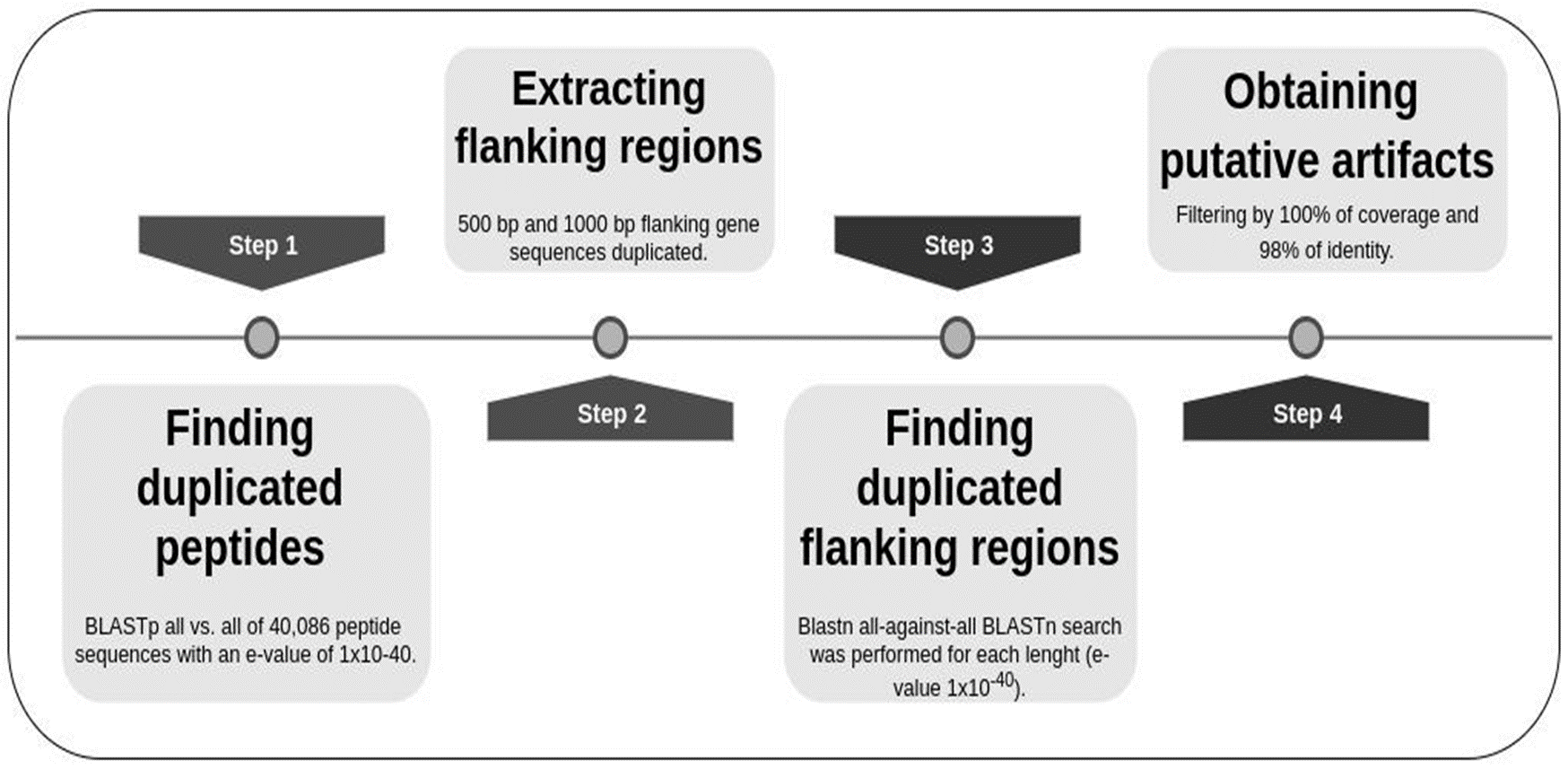


**Fig. S1: Solving excess heterozygosity**

Pipeline to detect putative artifacts duplicated in the new assembly of *Ae. albopictus*.


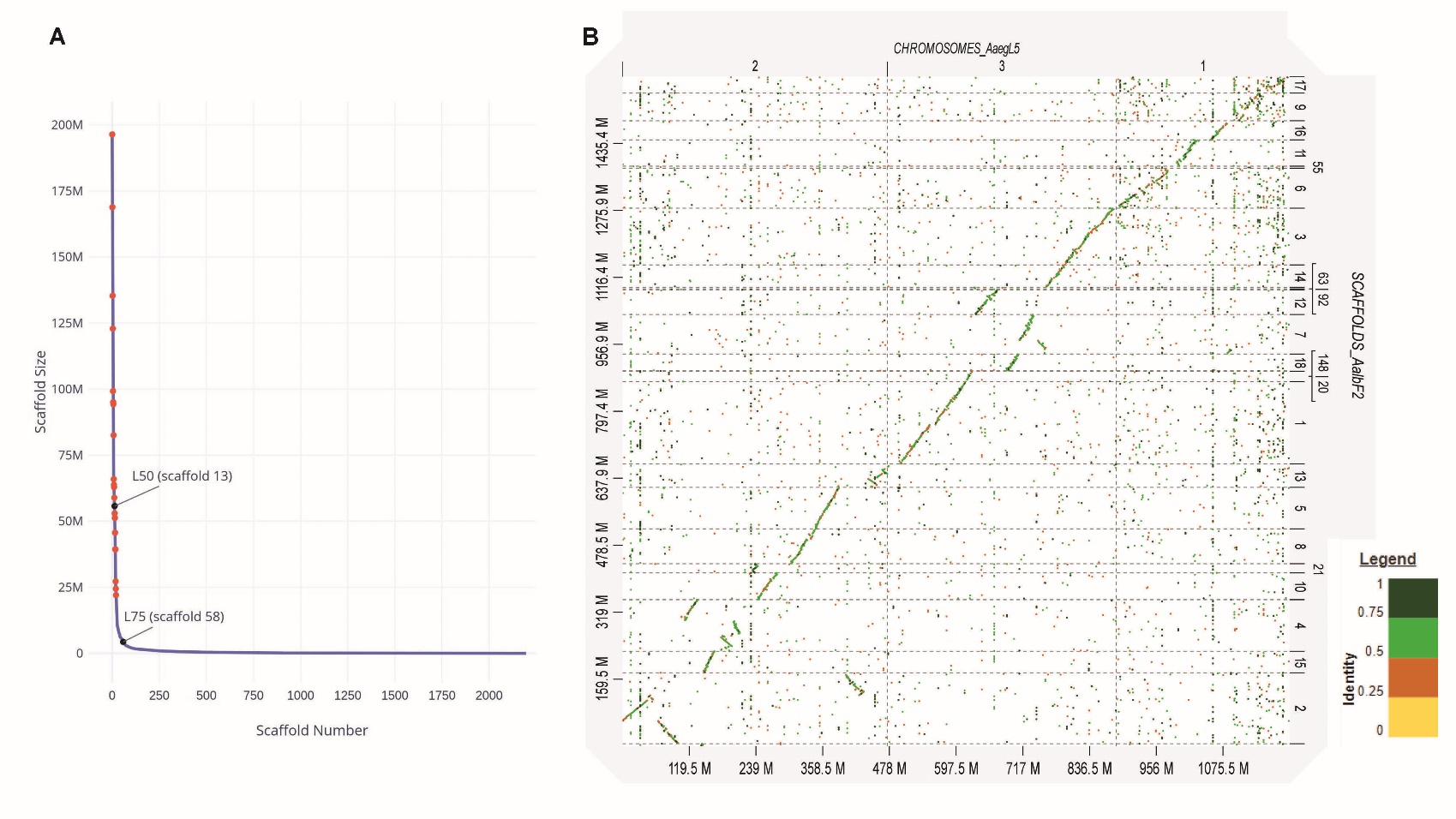


**Fig. S2: Correspondence between Ae. aegypti and Ae. albopictus**

**A)** AalbF2 scaffolds distributed by size. Red dots indicate the 19 scaffolds that have been mapped by FISH; the L50 and L75 are also shown. **B)** Mapping on *Ae. aegypti* chromosomes the 18 largest *Ae. albopictus* scaffolds, including those anchoed to the chromosomes by *in situ* hybridization.


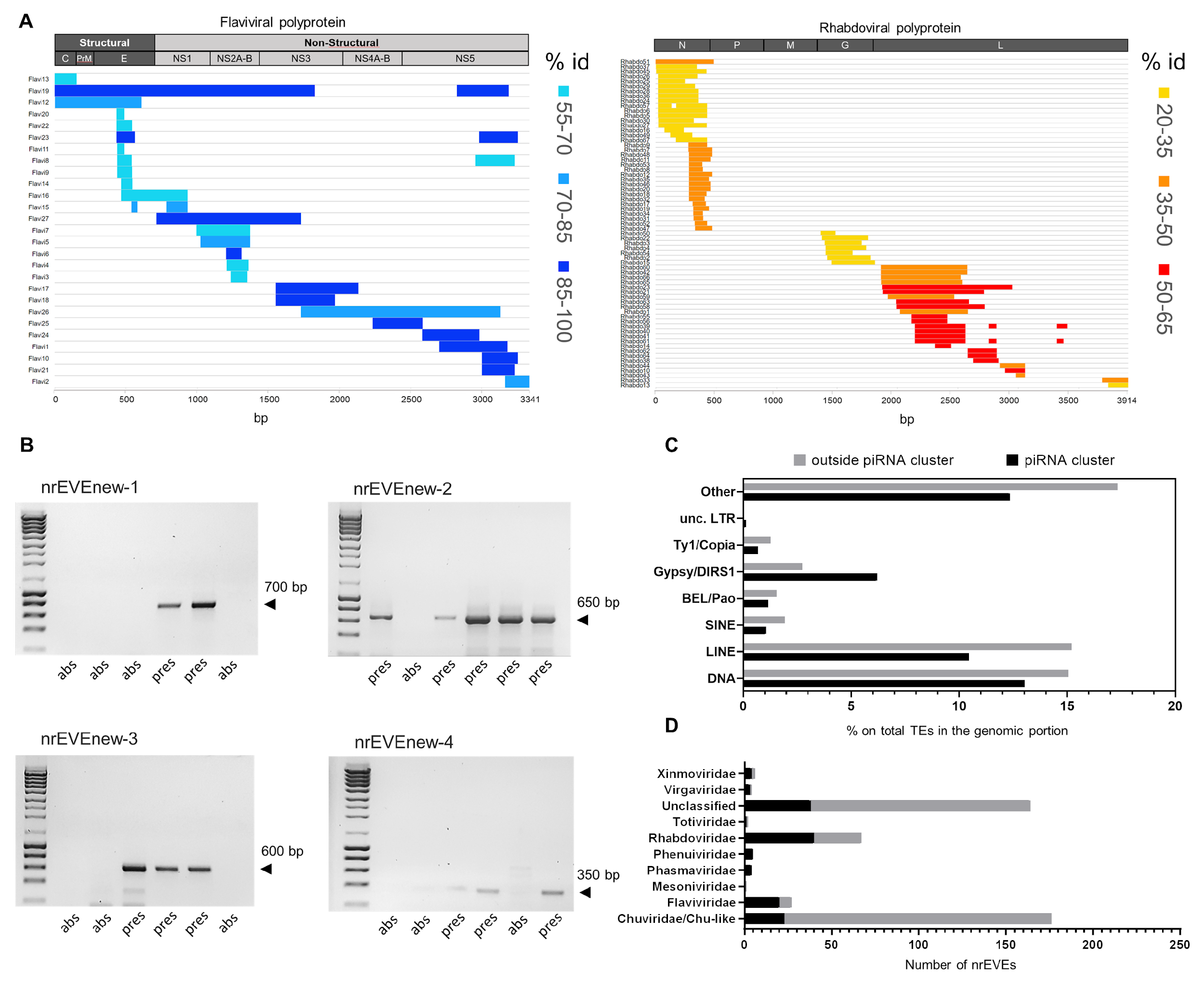

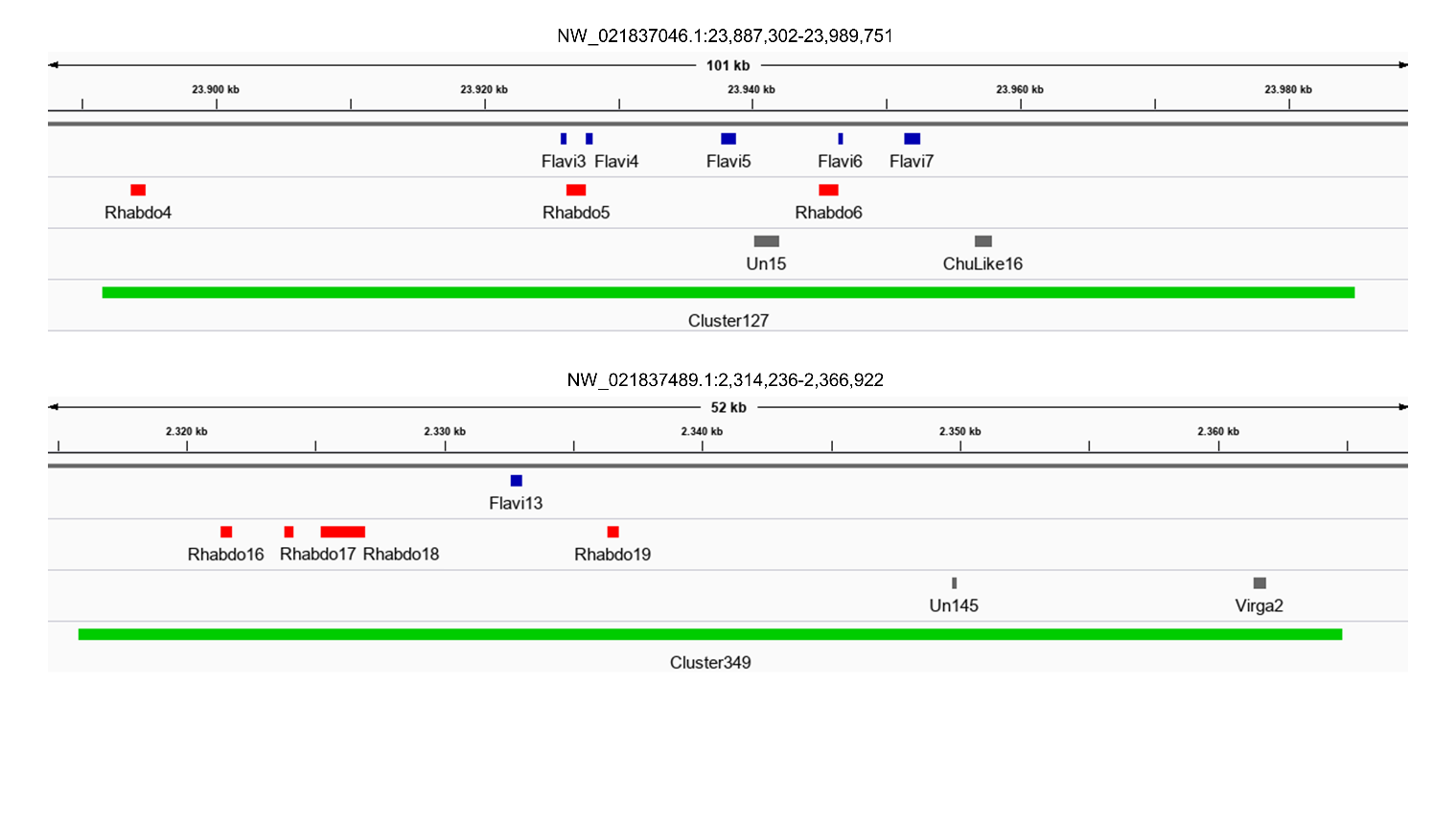


**E**

**Fig. S3: Viral integrations of the Ae. albopictus genome**

**A)** Juxtaposing of Flavi- and Rhabdo-EVEs to a scheme of the corresponding viral family genome structure. Viral integrations are color coded based on their percentage of identity to their most similar virus as shown in Table S6. **B)** PCR amplification of the novel nrEVEs in single wild-collected mosquitoes. **C)** Percentage of each TE category on the total genome content in and out piRNA clusters. **D)** Distribution of TEs (upper) and nrEVEs (lower) outside (gray) or within (black) piRNA clusters. In the lower panel, the X axis shows the number of viral integrations for each family as shown on the Y axis. **E)** IGV visualization of two nrEVEs clusters containing viral integrations derived from *Flaviviridae*, *Rhabdoviridae* and other viral families. The nrEVEs clusters are located on scaffolds NW_021837046.1 and NW_021837489.1 and are both embedded in piRNA clusters (i.e. piRNA cluster127 and 349, respectively).


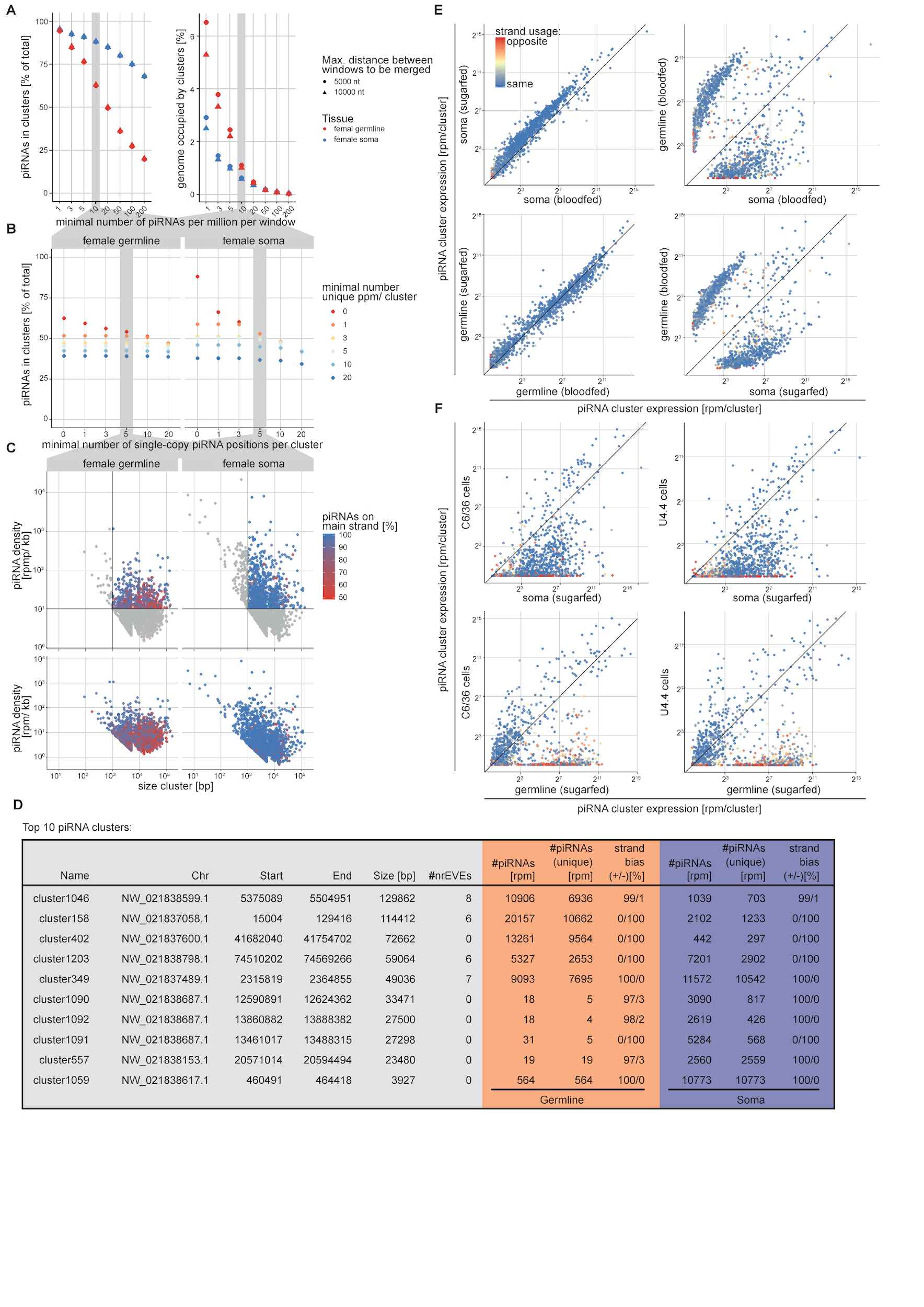


**Fig. S4: piRNA cluster annotation**

**A-C)** Impact of various arbitrary thresholds on cluster annotation (see materials and methods). Influence of **A)** the requirement for minimal number of piRNAs per 5 kb window, and the maximum distance between windows to be merged, or **B)** the requirement for a minimal coverage of uniquely mapping piRNAs, and for the number of single-loci piRNA positions on the fraction of piRNAs incorporated in clusters (left panels), or the fraction of the genome covered by clusters (right panels). **C)** Scatterplots indicating the sizes and piRNA densities of annotated clusters, with clusters falling below the minimal length and density requirement highlighted with shades. Colors display the bias of piRNA expression towards one dominant strand (with 50% corresponding to an unbiased piRNA expression from both strands, dual-strand clusters). rpmp, reads per million mapped piRNAs. For comparative reasons, cluster density when normalizing to total small RNAs (rpm, piRNA reads per million mapped reads) is indicated in the lower panels. **D)** Top ten piRNA clusters with at least 10 % of piRNAs being uniquely mapping. Clusters were ranked according to the average expression in soma and germline. **E, F)** log2 expression of piRNA clusters in different tissues **E)**, or cell lines **F)**. A pseudo-count of one was added to plot values of zero. Colors indicate the degree of accordance of the strand bias between both tissues.

**
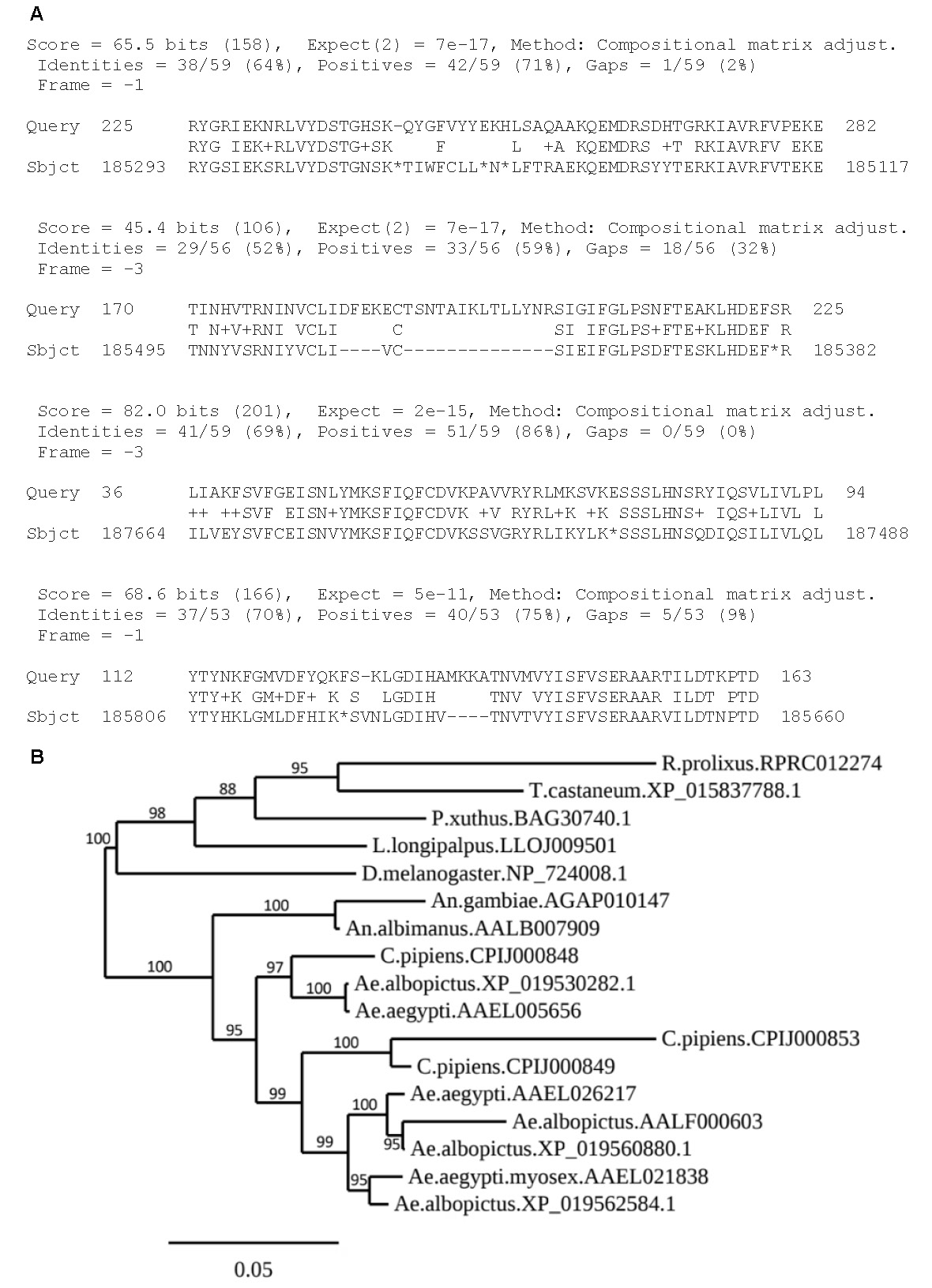
**

**Fig. S5: Insights into the M locus of Ae. albopictus
A)** Alignment between the *Ae. albopictus* NIX protein query sequence (XP_019525102) and a duplicated copy in contig NW_021838423.1. Note that the *Ae. albopictus* nix gene encoding XP_019525102 is also located in contig NW_021838423.1 from position 209080 to 210622.

**B)** PhyML Tree shows inferred phylogeny of *Ae. albopictus* and select mosquito myosin family deduced protein sequences. Species names are followed by sequence ID. The *Ae. albopictus* sequence XP_019562584.1 appears to be the ortholog to *Ae. aegypti* *myo-sex* that is located in the M-locus and involved in male flight (*38*, *48*, *55*).


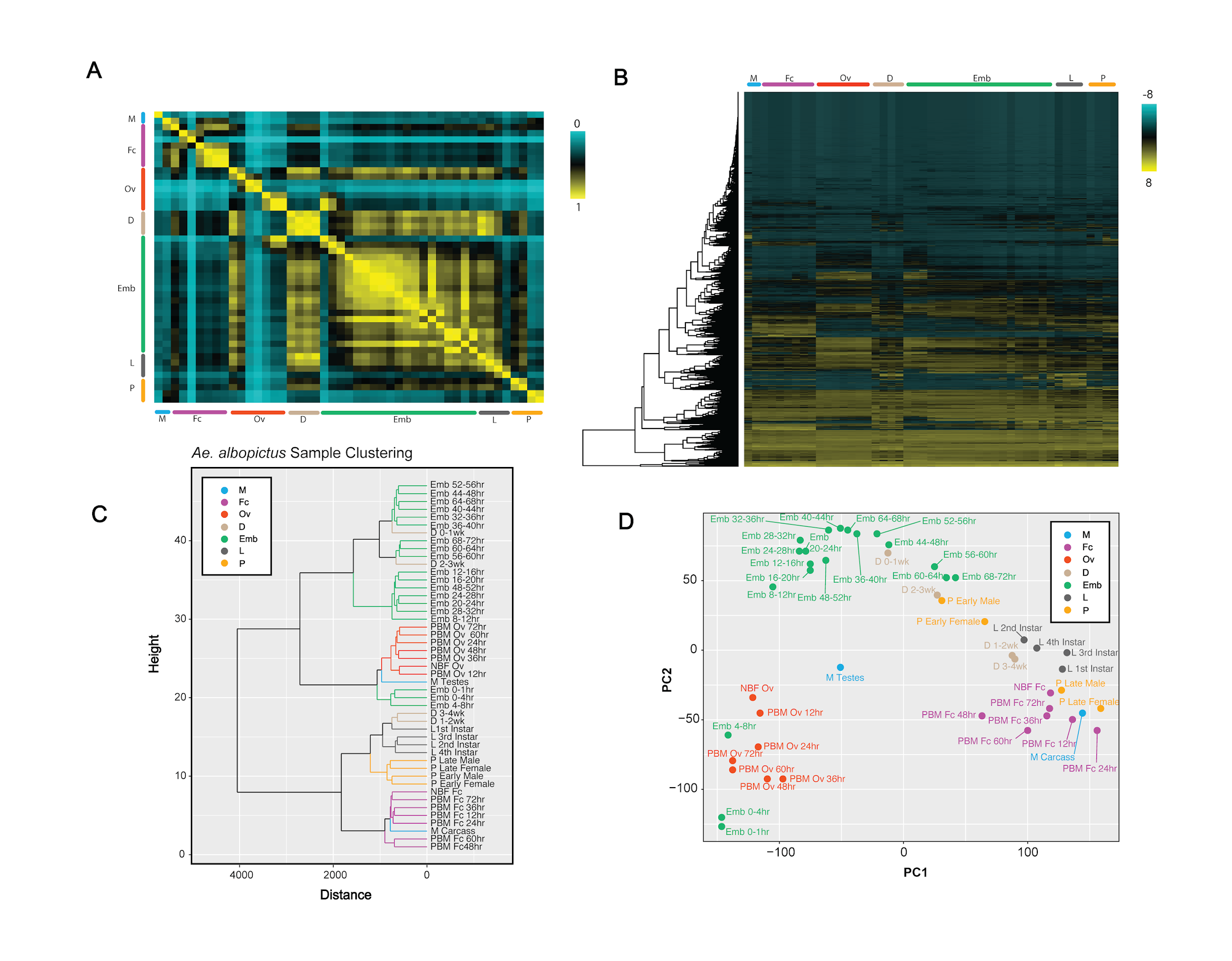


**D**

**C**

**B**

**A**

**Fig. S6: Global dynamics of gene expression**

**A)** Correlation (Pearson *r*) matrix of all RNA-seq timepoints for all known *Ae. albopictus* genes. **B)** Hierarchical clustering heat map of *Ae. albopictus* genes across all developmental stages. TPM values were log2(x+1) transformed and were scaled to plot the z-scores. **C)** Dendrogram of *Ae. albopictus* samples clustering similar life stages closer together. Plot depicts the close relationship between all developmental samples. **D)** PCA clustering of *Ae. albopictus* samples depicts clustering of life stages who show close similarity. PCA plot is in agreement with clustering dendrogram.
