# Supplementary material for: Improved reference genome of the arboviral vector Aedes albopictus
Source: Genome Biol. 2020 Aug 26;21:215. doi: 10.1186/s13059-020-02141-w (PMC7448346; doi:10.1186/s13059-020-02141-w)
Supplement: Supplementary file 2 — Additional file 2: Document containing supplementary tables S1 to S8. [file 13059_2020_2141_MOESM2_ESM.docx]

Additional File 2 for

Improved reference genome of the arboviral vector *Aedes albopictus*

Umberto Palatini, Reem A. Masri, Luciano V. Cosme, Sergey Koren, Françoise Thibaud-Nissen, James K. Biedler, Flavia Krsticevic, J. Spencer Johnston, Rebecca Halbach, Jacob E. Crawford, Igor Antoshechkin, Anna-Bella Failloux, Elisa Pischedda, Michele Marconcini, Jay Ghurye, Arang Rhie, Atashi Sharma, Dmitry A. Karagodin, Jeremy Jenrette, Stephanie Gamez, Pascal Miesen, Patrick Masterson, Adalgisa Caccone, Maria V. Sharakhova, Zhijian Tu, Philippos A. Papathanos, Ronald P. Van Rij, Omar S. Akbari, Jeffrey Powell, Adam M. Phillippy, Mariangela Bonizzoni

Correspondence to: [m.bonizzoni@unipv.it](mailto:m.bonizzoni@unipv.it)

**Additional file 1 includes supplementary tables S1 to S8**

**Contents:**

Table S1: Assembly statistics and validation.

Table S2: Transposable elements in AalbF2.

Table S3: Position of probes used for in situ hybridization.

Table S4: Scaffolds mapping on chromosomes.

Table S5: Association between AaloF1 nrEVEs and AalbF2 nrEVEs.

Table S6: nrEVEs alternative haplotypes.

Table S7: PCR primers used to confirm newly identified viral integrations.

Table S8: Expansion of PPO genes.

**Table S1: Assembly statistics and validation**

|  | **AaloF1** | **AalbF2** |
| --- | --- | --- |
| **Assembly Statistics** | | |
| Total assembly size | 1,923,476,627 bp | 2,538,387,871 bp |
| GC (%) | 40.05% | 40.40% |
| N. contigs | 355061 | 5556 |
| N. scaffolds | 154782 | 2197 |
| Scaffold N50 | 195,500 bp | 55,702,539 bp |
| L50^1^ | 2578 | 13 |
| Scaffold N75 | 198,159 bp | 4,348,337 bp |
| L75^1^ | 5433 | 58 |
| Max scaffold length | 1,305 Mb | 196,395 Mb |
| Percentage of the genome in scaffold > 50kb | 84.86% | 99.53% |
| **BUSCO Statistics** | | |
| Complete BUSCOs (C) | 2620 (93.6%) | 2610 (93.2%) |
| Complete and single-copy BUSCOs (S) | 1984 (70.9 %) | 2218 (79.2%) |
| Complete and duplicated BUSCOs (D) | 636 (22.7%) | 392 (14.0%) |
| Fragmented BUSCOs (F) | 94 (3.4%) | 70 (2.5%) |
| Missing BUSCOs (M) | 85 (3.0%) | 119 (4.3%) |
| Total BUSCO groups searched | 2799 (100%) | 2799 (100%) |
| **Barrnap Statistics** |  |  |
| Predicted rRNA genes | 22^2^ | 484^2^ |
| **Genome alignment Statistics^3^** | | |
| Alignment rate | 82.76% | 84.85% |
| Properly paired reads | 63.75% | 67.15% |
| Both Reads mapped | 65.00% | 72.53% |
| Singletons | 17.76% | 12.32% |
| **Transcriptome Data Statistics^4^** | | |
| Alignment rate | 82.04 ± 5.18% | 86.54 ± 4.57% |
| Properly paired reads | 70.92 ± 4.72% | 78.49 ± 4.22% |
| Both Reads mapped | 76.37 ± 4.98% | 82.02 ± 4.33% |
| Singletons | 5.68 ± 0.57% | 4.52 ± 0.40% |

Data table listing, comparatively between AalbF2 and AaloF1, assembly, BUSCO and Barrnap statistics; genome and transcriptome alignment rates.

^1^L50 and L75 are the smallest number of contigs whose length makes up 50% and 75% of the genome size, respectively.

^2^Total number of predicted rRNA genes. Estimated copy number of rDNA in haploid Ae. albopictus genome is 430 (8)

^3^Alignment of 16 singly-sequences Foshan mosquitoes (9) using MagicBlast (67).

^4^Alignment of published RNA-seq data (BioProject PRJNA475859) using Hisat2 (68).

**Table S2: Transposable elements in AalbF2**

**A)** Transposable elements (TEs) annotated by Repeatmasker in the AalbF2 genome; **B)** Comparison between the TE repertoire of the AalbF2, AaloF1 and AaegL5 genome assemblies.

**A**

|  | Number of elements | Occupancy (bp) | Percentage of sequence | |
| --- | --- | --- | --- | --- |
| **Retroelements** | **1430646** | **575946434** | | **22.69%** |
| SINEs: | 208123 | 48743703 | | 1.92% |
| Penelope | 103794 | 18576486 | | 0.73% |
| LINEs: | 866027 | 384927593 | | 15.16% |
| CRE/SLACS | 0 | 0 | | 0.00% |
| L2/CR1/Rex | 81301 | 32399143 | | 1.28% |
| R1/LOA/Jockey | 320615 | 167497304 | | 6.6% |
| R2/R4/NeSL | 8369 | 2745389 | | 0.11% |
| RTE/Bov-B | 317328 | 149941249 | | 5.91% |
| L1/CIN4 | 9665 | 4479453 | | 0.18% |
| **LTR elements:** | **356496** | **142275138** | | **5.6%** |
| BEL/Pao | 93848 | 39116362 | | 1.54% |
| Ty1/Copia | 81033 | 32119258 | | 1.27% |
| Gypsy/DIRS1 | 180988 | 69910987 | | 2.75% |
| Retroviral | 0 | 0 | | 0.00% |
|  |  |  | |  |
| **DNA transposons** | **1485602** | **382032412** | | **15.05%** |
| hobo-Activator | 121429 | 30954936 | | 1.22% |
| Tc1-IS630-Pogo | 113870 | 30939226 | | 1.22% |
| En-Spm | 0 | 0 | | 0.00% |
| MuDR-IS905 | 0 | 0 | | 0.00% |
| PiggyBac | 9040 | 2702468 | | 0.11% |
| Tourist/Harbinger | 15859 | 3420295 | | 0.13% |
| Other (Mirage, P-element, Transib) | 7028 | 1202716 | | 0.05% |
|  |  |  | |  |
| Rolling-circles | 67687 | 19651620 | | 0.77% |
|  |  |  | |  |
| Unclassified: | 1721247 | 439014757 | | 17.3% |
|  |  |  | |  |
| Total interspersed repeats: |  | 1396993603 | | 55.03% |
|  |  |  | |  |
| Satellites: | 382834 | 164062446 | | 6.46% |
| Simple repeats: | 562796 | 193327114 | | 7.62% |
| Low complexity: | 22198 | 1161877 | | 0.05% |

* most repeats fragmented by insertions or deletions have been counted as one element

RepeatMasker Combined Database: Dfam_3.1

run with rmblastn version 2.10.0+

**B**

|  | **AalbF2** | **AaloF1** | **AaegL5** |
| --- | --- | --- | --- |
| **DNA** | 15.05% | 8.52% | 15.06% |
| **LINE** | 15.16% | 34.67% | 16.09% |
| **SINE** | 1.92% | 0.07% | 1.16% |
| **LTR** | 5.60% | 16.21% | 11.66% |
| **Other** | 17.30% | 8.85% | 10.88% |
| **TOTAL TEs** | 55.03% | 68.33% | 54.85% |

**Table S3: Position of probes used for in situ hybridizatio**

Transcript positions in the *Ae. albopictus* and *Ae. aegypti* (AaegL5) genome assemblies and in the chromosomes of *Ae. albopictus*. Transcripts are indicated by accession number.

| **Scaffold ID (AalbF2)** | **Transcript (C6/36 assembly)** | **Scaffold** | **Start position** | **Location** | **Location in *Ae. aegypti*** | **Status with respect to *Ae. aegypti*** |
| --- | --- | --- | --- | --- | --- | --- |
| NW_021837045.1 | XM_019703761.1 | 1 | 141,912 | 3p34 | 3p:24,232,751 | Consistent |
| NW_021837045.1 | XM_019687494.1 | 1 | 78,206,953 | 3p44 | 3p:67,490,043 | Consistent |
| NW_021837045.1 | XM_019689702.1 | 1 | 102,316,324 | 3p34 | 3p:89,374,076 | Consistent |
| NW_021837045.1 | XM_019684861.1 | 1 | 115,572,240 | 3p32 | 3p:94,611,521 | Consistent |
| NW_021837045.1 | XM_019682463.1 | 1 | 129,449,579 | 3p32 | 3p:102,088,493 | Consistent |
| NW_021837045.1 | XM_019690818.1 | 1 | 132,846,558 | 3p32 | 3p:104,272,700 | Consistent |
| NW_021838153.1 | XM_019691989.1 | 2 | 8,394,996 | 2p35 | 2p:93,350,227 | Consistent |
| NW_021838153.1 | XM_019675272.2 | 2 | 32,875,176 | 2p32 | 2p:76,267,897 | Consistent |
| NW_021838153.1 | XM_019682530.1 | 2 | 129,936,557 | 2q44 | 2q:422,726,667 | Consistent |
| NW_021838465.1 | XM_019704755.1 | 3 | 18,369,974 | 3q31 | 3q:325,406,225 | Consistent |
| NW_021838465.1 | XM_019701500.1 | 3 | 114,597,945 | 3q43 | 3q:392,144,268 | Consistent |
| NW_021838576.1 | XM_019675405.1 | 4 | 15,276,711 | 2p22 | 2p:126,200,520 | inversion |
| NW_021838576.1 | XM_019673127.1 | 4 | 24,157,064 | 2p25 | 2p:121,906,299 | Consistent |
| NW_021838576.1 | XM_019677471.1 | 4 | 24,157,064 | 2p25 | 2p:121,906,299 | Consistent |
| NW_021838576.1 | XM_019681895.1 | 4 | 53,051,265 | 2p12 | 2p:198,995,083 | Consistent |
| NW_021838576.1 | XM_019698846.1 | 4 | 68,135,981 | 2p12 | 2p:203,995,757 | Consistent |
| NW_021838576.1 | XM_019698741.1 | 4 | 77,060,065 | 2p12 | 2q:210,256,925 | Consistent |
| NW_021838687.1 | XM_019703202.1 | 5 | 2,060,636 | 2q34 | 2q:387,886,655 | Consistent |
| NW_021838687.1 | XM_019694026.1 | 5 | 21,213,967 | 2q33 | 2q:378,098,440 | Consistent |
| NW_021838687.1 | XM_019694057.1 | 5 | 23,813,996 | 2q33 | 2q:378,806,902 | Consistent |
| NW_021838687.1 | XM_019694256.1 | 5 | 56,553,773 | 2q31 | 2q:360,884,653 | Consistent |
| NW_021838798.1 | XM_019696654.1 | 6 | 28,914,960 | 1p25 | 1p:73,017,099 | Consistent |
| NW_021838798.1 | XM_019706593.1 | 6 | 69,371,979 | 1p34 | 1p:27,332,552 | Consistent |
| NW_021838909.1 | XM_019676257.1 | 7 | 81,139,711 | 3q14 | 3q:255,525,249 | Consistent |
| NW_021838909.1 | XM_019705822.1 | 7 | 8,146,758 | 1q21 | 1q:203,878,017 | Consistent |
| NW_021839020.1 | XM_019670988.1 | 8 | 23,795,330 | 2q24 | 2q:316,771,716 | Consistent |
| NW_021839020.1 | XM_019695499.1 | 8 | 60,172,454 | 2q24 | 2q:336,171,722 | Consistent |
| NW_021839130.1 | XM_019686202.1 | 9 | 16,379,504 | 1q31 | 1q:242,666,370 | Consistent |
| NW_021839130.1 | XM_019686203.1 | 9 | 16,379,504 | 1q31 | 1q:242666370 | Consistent |
| NW_021839130.1 | XM_019685481.1 | 9 | 58,786,903 | 1q33 | 1q:223,480,890 | Consistent |
| NW_021837267.1 | XM_019704970.1 | 12 | 109,742 | 3p11 | 3p:178,234,261 | Consistent |
| NW_021837267.1 | XM_019674355.1 | 12 | 4,413,385 | 3p13 | 3p:160,039,183 | consistent |
| NW_021837267.1 | XM_019677377.1 | 12 | 47,026,645 | 3p12 | 3p:189,773,252 | Consistent |
| NW_021837378.1 | XM_019674838.1 | 13 | 20,304,874 | 2q46 | 2q:439,707,785 | Consistent |
| NW_021837489.1 | XM_019699815.1 | 14 | 489,131 | 3q23 | 3q:313,741,920 | Consistent |
| NW_021837489.1 | XM_019707152.1 | 14 | 49,492,391 | 3q23 | 3q:286,848,677 | Consistent |
| NW_021837600.1 | XM_019691051.1 | 15 | 49,408,719 | 2p22 | 2p:147,096,234 | Consistent |
| NW_021837711.1 | XM_019698166.1 | 16 | 2,841,814 | 1q12 | 1q:171,186,806 | Consistent |
| NW_021837822.1 | XM_019698650.1 | 17 | 27,654,978 | 1q44 | 1q:292,319,975 | Consistent |
| NW_021837931.1 | XM_019682829.1 | 18 | 20,015,332 | 3q11 | 3q:225,565,535 | Consistent |
| NW_021838154.1 | XM_019699074.1 | 20 | 2,199,384 | 3p21 | 3p:142,620,468 | Consistent |
| NW_021838154.1 | XM_019697550.1 | 20 | 22,883,182 | 3p14 | 3p:151,341,908 | Consistent |
| NW_021838233.1 | XM_019703990.1 | 21 | 12,395,103 | 2q12, CM1,3 | 2q: 238,557,210 | Consistent |
| NW_021838233.1 | XM_019703499.1 | 21 | 15,046,841 | 2q11 | 2q:241,488,879 | Consistent |
| NW_021838665.1 | XM_020077126.1 | 48 | 3,448,080 | 2p24 | 2p:143,591,914 | Consistent |
| NW_021838743.1 | XM_019702030.1 | 55 | 2,960,159 | 3q34 | 3q:362,330,504 | Consistent |
| NW_021838832.1 | XM_019675517.1 | 63 | 1,551,340 | 3p13 | 3p:156,723,676 | Consistent |
| NW_021839153.1 | XM_019696917.1 | 92 | 864,056 | 3q13 | 3q:234,093,089 | Consistent |
| NW_021837578.1 | XM_019691611.1 | 148 | 98,118 | 2q26 | 2q:344,922,562 | Consistent |

**Table S4: Scaffolds mapping on chromosomes**

Bioinformatically mapping the first 58 scaffolds (L75) to the chromosomes of *Aedes albopictus* using mapping alignments to *Aedes aegypti* chromosomes. Support for the analyses comes from the correspondence between bioinformatic-based and *in situ* mapped scaffolds.

| **Scaffold ID** | **Scaffold Number** | **FISH-mapped chromosome** | **DGENIES target chromosome*** | **Scaffold Len** | | **Q-start** | **Q-stop** | **T-len** | **T-start** | **T-stop** |
| --- | --- | --- | --- | --- | --- | --- | --- | --- | --- | --- |
| NW_021837045.1 | 1 | 3p32 | 3 | 196395033 | | 4836 | 196394362 | 409777670 | 818751 | 409343898 |
| NW_021838153.1 | **2** | 2p32 | 2 | 168827982 | | 97908 | 168459680 | 474425716 | 31540 | 472179123 |
| NW_021838465.1 | 3 | 3q31 | 3 | 135305655 | | 75172 | 135305593 | 409777670 | 282374 | 409231705 |
| NW_021838576.1 | 4 | 2p12 | 2 | 122869687 | | 65214 | 122845322 | 474425716 | 709956 | 472518817 |
| NW_021838687.1 | 5 | 2q31 | 2 | 99254364 | | 7308 | 99184464 | 474425716 | 567355 | 472224827 |
| NW_021838798.1 | 6 | 1p25 | 1 | 95072813 | | 14066 | 95026473 | 310827022 | 740768 | 309548875 |
| NW_021838909.1 | 7 | 3q14** | 3 | 94263231 | | 94841 | 94255626 | 409777670 | 1163769 | 408456253 |
| NW_021839020.1 | 8 | 2q24 | 2 | 82511891 | | 8220 | 82459432 | 474425716 | 188809 | 472227243 |
| NW_021839130.1 | 9 | 1q31 | 1 | 65883261 | | 50050 | 65882908 | 310827022 | 1159548 | 309433748 |
| NW_021837046.1 | 10 |  | 2 | 63746563 | | 2478 | 63720061 | 474425716 | 709956 | 472224467 |
| NW_021837156.1 | 11 |  | 1 | 62838808 | | 42717 | 62811988 | 310827022 | 2319182 | 310163075 |
| NW_021837267.1 | 12 | 3p11 | 3 | 58853413 | | 33637 | 58828551 | 409777670 | 930522 | 408972797 |
| NW_021837378.1 | 13 | 2q46 | 2 | 55702539 | | 29171 | 55692883 | 474425716 | 2001175 | 474378467 |
| NW_021837489.1 | 14 | 3q23 | 3 | 52942089 | | 77635 | 52901422 | 409777670 | 235555 | 409232253 |
| NW_021837600.1 | 15 | 2p22 | 2 | 51173165 | | 109619 | 51172994 | 474425716 | 1572995 | 472518811 |
| NW_021837711.1 | 16 | 1q12 | 1 | 45635565 | | 10781 | 45246087 | 310827022 | 2318946 | 310009407 |
| NW_021837822.1 | 17 | 1q44 | 1 | 39427437 | | 36112 | 39206771 | 310827022 | 2426604 | 310163813 |
| NW_021837931.1 | 18 | 3q11 | 3 | 39279557 | | 45612 | 39141352 | 409777670 | 1508614 | 408454682 |
| NW_021838042.1 | 19 |  | 2 | 27194535 | | 30173 | 27178416 | 474425716 | 2994068 | 472227746 |
| NW_021838154.1 | 20 | 3p14 | 3 | 24405320 | | 67845 | 24370121 | 409777670 | 1914801 | 407228286 |
| NW_021838233.1 | 21 | 2q11 | 2 | 21999845 | | 79 | 21990515 | 474425716 | 2937402 | 472178394 |
| NW_021838343.1 | 22 |  | 2 | 21387314 | | 549 | 21371175 | 474425716 | 1506814 | 472227710 |
| NW_021838388.1 | 23 |  | 2 | 15820435 | | 5307 | 15769690 | 474425716 | 2937575 | 472222761 |
| NW_021838399.1 | 24 |  | 2 | 14680195 | | 484 | 14678688 | 474425716 | 6939110 | 472518817 |
| NW_021838410.1 | 25 |  | 1 | 12700773 | | 40729 | 12687724 | 310827022 | 1782533 | 309430400 |
| NW_021838421.1 | 26 |  | 1 | 11967382 | | 13254 | 11790126 | 310827022 | 3841683 | 309431875 |
| NW_021838432.1 | 27 |  | 3 | 11907018 | | 114409 | 11812395 | 409777670 | 1508614 | 395666574 |
| NW_021838443.1 | 28 |  | 2 | 10504575 | | 41351 | 10468552 | 474425716 | 13387261 | 472518817 |
| NW_021838454.1 | 29 |  | 2 | 10455035 | | 4631 | 10406668 | 474425716 | 13387261 | 467193453 |
| NW_021838466.1 | 30 |  | 2 | 10003331 | | 24750 | 9997291 | 474425716 | 1407962 | 470318076 |
| NW_021838477.1 | 31 |  | 2 | 8923808 | | 3285 | 8794727 | 474425716 | 197573 | 472177009 |
| NW_021838488.1 | 32 |  | 3 | 8738692 | | 2196 | 8738198 | 409777670 | 7429763 | 409232253 |
| NW_021838499.1 | 33 |  | 1 | 8696722 | | 112 | 8474377 | 310827022 | 23506573 | 310061255 |
| NW_021838510.1 | 34 |  | 3 | 8296403 | | 28976 | 8271757 | 409777670 | 44516148 | 409232286 |
| NW_021838521.1 | 35 |  | 1 | 8124246 | | 43774 | 8103026 | 310827022 | 10519127 | 309421208 |
| NW_021838532.1 | 36 |  | 2 | 7801424 | | 88675 | 7709837 | 474425716 | 2994046 | 464470419 |
| NW_021838543.1 | 37 |  | 1 | 7739647 | | 6875 | 7688847 | 310827022 | 6436673 | 300445293 |
| NW_021838554.1 | 38 |  | 3 | 7645035 | | 631 | 7644566 | 409777670 | 1915529 | 407252759 |
| NW_021838565.1 | 39 |  | 1 | 7506809 | | 204 | 7443195 | 310827022 | 15938023 | 307812612 |
| NW_021838577.1 | 40 |  | 3 | 7114296 | | 197923 | 7010106 | 409777670 | 1914801 | 408454655 |
| NW_021838588.1 | 41 |  | 2 | 7078098 | | 44447 | 6848836 | 474425716 | 3271702 | 472040095 |
| NW_021838599.1 | 42 |  | 3 | 6377459 | | 61750 | 6157821 | 409777670 | 1508614 | 397802913 |
| NW_021838610.1 | 43 |  | 1 | 6209708 | | 170821 | 6160254 | 310827022 | 5576272 | 309430965 |
| NW_021838621.1 | 44 |  | 1 | 6075262 | | 72854 | 5967581 | 310827022 | 2424055 | 306235135 |
| NW_021838632.1 | 45 |  | 1 | 6042954 | | 25107 | 5988754 | 310827022 | 4643554 | 309430216 |
| NW_021838643.1 | 46 |  | 2 | 6008651 | | 7869 | 5960461 | 474425716 | 16012949 | 472224467 |
| NW_021838654.1 | 47 |  | 3 | 5997344 | | 9512 | 5931893 | 409777670 | 7649196 | 407228286 |
| NW_021838665.1 | 48 | 2p24 | 2 | 5928163 | | 30231 | 5921784 | 474425716 | 17069773 | 472224805 |
| NW_021838676.1 | 49 |  | 1 | 5616786 | | 4674 | 5595548 | 310827022 | 13708366 | 309430965 |
| NW_021838688.1 | 50 |  | 1 | 5603677 | | 11 | 5565027 | 310827022 | 453107 | 305074731 |
| NW_021838699.1 | 51 |  | 1 | 5568893 | | 103110 | 5483763 | 310827022 | 13711300 | 307812681 |
| NW_021838710.1 | 52 |  | 1 | 5428130 | | 61260 | 5279199 | 310827022 | 17362205 | 304230677 |
| NW_021838721.1 | 53 |  | 2 | 4868804 | | 19270 | 4821996 | 474425716 | 12717555 | 468601915 |
| NW_021838732.1 | 54 |  | 1 | 4850854 | | 1455 | 4768656 | 310827022 | 16239465 | 306128849 |
| NW_021838743.1 | 55 | 3q34 | 1 | 4826477 | | 29747 | 4762033 | 310827022 | 14142692 | 307812680 |
| NW_021838754.1 | 56 |  | 1 | 4637188 | | 35013 | 4606591 | 310827022 | 15936991 | 307490695 |
| NW_021838765.1 | 57 |  | 2 | | 4606087 | 17708 | 4515852 | 474425716 | 188809 | 472177152 |
| NW_021838776.1 | 58 |  | 2 | | 4348337 | 12679 | 4343083 | 474425716 | 5578639 | 466433958 |

*Mapped versus Ae. aegypti AegL5 assembly chromosomes

** FISH probes suggested either 1q21 or 3q14. Bioinformatical analyses confirmed Chromosome 3

**Table S5: Association between AaloF1 nrEVEs and AalbF2 nrEVEs**

Data table listing the viral integrations annotated in AaloF1 and AalbF2 assembly.

|  | **AalbF2** |  |
| --- | --- | --- |
| **AaloF1** | **ID > 99%** | **ID < 99%*** |
| AlbFlavi1 | 0 | Flavi11, Flavi9, Flavi8, Flavi20, Flavi22 |
| AlbFlavi10 | Flavi26, Flavi24 | 0 |
| AlbFlavi12_17 | Flavi8 | Flavi10, Flavi21, Flavi9, Flavi11, Flavi23, Flavi22, Flavi23, Flavi20 |
| AlbFlavi18 | 0 | 0 |
| AlbFlavi19 | 0 | 0 |
| AlbFlavi2 | Flavi12 | 0 |
| AlbFlavi20 | 0 | 0 |
| AlbFlavi22 | 0 | Flavi4, Flavi7, Flavi3, Flavi6, Flavi5 |
| AlbFlavi23 | Flavi6, Flavi7 | Flavi4, Flavi3, Flavi5 |
| AlbFlavi24 | Flavi27 | 0 |
| AlbFlavi25 | 0 | Flavi3, Flavi7 |
| AlbFlavi26 | 0 | Flavi7, Flavi5, Flavi4, Flavi3, Flavi6 |
| AlbFlavi27 | 0 | Flavi4, Flavi7, Flavi3, Flavi6, Flavi5 |
| AlbFlavi28 | 0 | 0 |
| AlbFlavi3 | Flavi12 | 0 |
| AlbFlavi31 | 0 | Flavi27 |
| AlbFlavi32 | 0 | Flavi27 |
| AlbFlavi33 | Flavi27 | 0 |
| AlbFlavi34 | 0 | Flavi27, Flavi26, Flavi17, Flavi18 |
| AlbFlavi36 | Flavi25, Flavi26 | 0 |
| AlbFlavi37 | 0 | Flavi7, Flavi6, Flavi3, Flavi5, Flavi4 |
| AlbFlavi38 | 0 | 0 |
| AlbFlavi39 | 0 | 0 |
| AlbFlavi4 | Flavi13 | 0 |
| AlbFlavi40 | 0 | 0 |
| AlbFlavi41 | Flavi15 | Flavi16 |
| AlbFlavi42 | Flavi5, Flavi7 | 0 |
| AlbFlavi6 | 0 | Flavi1 |
| AlbFlavi7 | 0 | Flavi1 |
| AlbFlavi8 | Flavi15 | Flavi16 |
| AlbRha1 | 0 | Rhabdo2 |
| AlbRha10 | 0 | 0 |
| AlbRha11 | Un144 | 0 |
| AlbRha12 | Rhabdo37, Rhabdo26 | Rhabdo30, Rhabdo25 |
| AlbRha14 | Xinmo3 | 0 |
| AlbRha15 | Rhabdo37, Rhabdo26 | Rhabdo30, Rhabdo25 |
| AlbRha18 | Rhabdo27 | 0 |
| AlbRha2 | Un151 | Un155 |
| AlbRha28 | 0 | Rhabdo28, Rhabdo24, Rhabdo29, Rhabdo36 |
| AlbRha3 | 0 | 0 |
| AlbRha32 | Un26, Un24 | 0 |
| AlbRha33 | 0 | Rhabdo4 |
| AlbRha36 | Rhabdo16 | 0 |
| AlbRha38 | 0 | 0 |
| AlbRha4 | Rhabdo55, Rhabdo56 | 0 |
| AlbRha41 | 0 | 0 |
| AlbRha42 | 0 | 0 |
| AlbRha43 | 0 | Rhabdo4 |
| AlbRha44 | 0 | 0 |
| AlbRha45 | 0 | Rhabdo15 |
| AlbRha48 | Rhabdo67 | Rhabdo49 |
| AlbRha49 | Rhabdo3 | 0 |
| AlbRha52 | Rhabdo21 | Rhabdo58, Rhabdo59 |
| AlbRha58 | 0 | Rhabdo4 |
| AlbRha62 | 0 | Rhabdo6, Rhabdo5 |
| AlbRha66 | Un2 | Rhabdo1 |
| AlbRha7 | 0 | 0 |
| AlbRha71 | 0 | Rhabdo6, Rhabdo5 |
| AlbRha73 | 0 | 0 |
| AlbRha74 | 0 | 0 |
| AlbRha79 | 0 | 0 |
| AlbRha80 | 0 | 0 |
| AlbRha83 | Rhabdo60, Rhabdo61, Rhabdo63, Rhabdo42, Rhabdo39, Rhabdo41, Rhabdo40 | 0 |
| AlbRha84 | Rhabdo60, Rhabdo42 | Rhabdo63, Rhabdo65, Rhabdo66 |
| AlbRha85 | Rhabdo38, Rhabdo40, Rhabdo62, Rhabdo41, Rhabdo39, Rhabdo61, Rhabdo64, | 0 |
| AlbRha87 | Rhabdo60, Rhabdo63, Rhabdo61, Rhabdo39, Rhabdo40, Rhabdo41, Rhabdo42 | Rhabdo66 |
| AlbRha88 | Rhabdo44 | 0 |
| AlbRha9 | Rhabdo36, Rhabdo28, Rhabdo24 | Rhabdo29 |
| AlbRha92 | 0 | Rhabdo67, Rhabdo49 |
| AlbRha94 | 0 | Rhabdo6, Rhabdo5 |
| AlbRha95 | Un26, Un24 | 0 |
| AlbRha96 | Rhabdo41, Rhabdo64, Rhabdo39, Rhabdo40, Rhabdo42, Rhabdo38, Rhabdo61, Rhabdo60, Rhabdo62, Rhabdo63 | Rhabdo66 |

*identity varied between 99-90%.

**Table S6: nrEVEs alternative haplotypes**

Number of alternative haplotypes found in the Ae. albopictus alternative assembly (GCA_006496715.1) for nrEVEs annotated in AalbF2.

| **nrEVE** | **Number of Haplotypes** |
| --- | --- |
| Canu_Flavi1 | 1 |
| Canu_Flavi10 | 1 |
| Canu_Flavi11 | 2 |
| Canu_Flavi12 | 3 |
| Canu_Flavi13 | 2 |
| Canu_Flavi14 | 1 |
| Canu_Flavi15 | 0 |
| Canu_Flavi16 | 1 |
| Canu_Flavi17 | 0 |
| Canu_Flavi18 | 0 |
| Canu_Flavi19 | 0 |
| Canu_Flavi2 | 0 |
| Canu_Flavi20 | 2 |
| Canu_Flavi21 | 0 |
| Canu_Flavi22 | 0 |
| Canu_Flavi23 | 1 |
| Canu_Flavi24 | 0 |
| Canu_Flavi25 | 0 |
| Canu_Flavi26 | 0 |
| Canu_Flavi27 | 0 |
| Canu_Flavi3 | 0 |
| Canu_Flavi4 | 2 |
| Canu_Flavi5 | 1 |
| Canu_Flavi6 | 3 |
| Canu_Flavi7 | 0 |
| Canu_Flavi9 | 0 |
| Canu_Flavi8 | 0 |
| Canu_Phasma1 | 2 |
| Canu_Phasma2 | 0 |
| Canu_Phasma3 | 0 |
| Canu_Phasma4 | 3 |
| Canu_Phenui1 | 0 |
| Canu_Phenui2 | 2 |
| Canu_Phenui3 | 2 |
| Canu_Phenui4 | 3 |
| Canu_Phenui5 | 0 |
| Canu_Rhabdo1 | 0 |
| Canu_Rhabdo10 | 0 |
| Canu_Rhabdo11 | 1 |
| Canu_Rhabdo12 | 2 |
| Canu_Rhabdo13 | 0 |
| Canu_Rhabdo14 | 0 |
| Canu_Rhabdo15 | 0 |
| Canu_Rhabdo16 | 4 |
| Canu_Rhabdo17 | 10 |
| Canu_Rhabdo18 | 1 |
| Canu_Rhabdo19 | 3 |
| Canu_Rhabdo2 | 1 |
| Canu_Rhabdo20 | 3 |
| Canu_Rhabdo21 | 0 |
| Canu_Rhabdo22 | 0 |
| Canu_Rhabdo23 | 1 |
| Canu_Rhabdo24 | 1 |
| Canu_Rhabdo25 | 0 |
| Canu_Rhabdo26 | 0 |
| Canu_Rhabdo27 | 0 |
| Canu_Rhabdo28 | 0 |
| Canu_Rhabdo29 | 1 |
| Canu_Rhabdo3 | 2 |
| Canu_Rhabdo30 | 1 |
| Canu_Rhabdo31 | 2 |
| Canu_Rhabdo32 | 1 |
| Canu_Rhabdo33 | 0 |
| Canu_Rhabdo34 | 2 |
| Canu_Rhabdo35 | 2 |
| Canu_Rhabdo36 | 1 |
| Canu_Rhabdo37 | 0 |
| Canu_Rhabdo38 | 3 |
| Canu_Rhabdo39 | 2 |
| Canu_Rhabdo4 | 1 |
| Canu_Rhabdo40 | 2 |
| Canu_Rhabdo41 | 2 |
| Canu_Rhabdo42 | 4 |
| Canu_Rhabdo43 | 0 |
| Canu_Rhabdo44 | 0 |
| Canu_Rhabdo45 | 2 |
| Canu_Rhabdo46 | 2 |
| Canu_Rhabdo47 | 3 |
| Canu_Rhabdo48 | 1 |
| Canu_Rhabdo49 | 0 |
| Canu_Rhabdo5 | 0 |
| Canu_Rhabdo50 | 0 |
| Canu_Rhabdo51 | 1 |
| Canu_Rhabdo52 | 3 |
| Canu_Rhabdo53 | 0 |
| Canu_Rhabdo54 | 1 |
| Canu_Rhabdo55 | 1 |
| Canu_Rhabdo56 | 1 |
| Canu_Rhabdo57 | 2 |
| Canu_Rhabdo58 | 0 |
| Canu_Rhabdo59 | 1 |
| Canu_Rhabdo6 | 2 |
| Canu_Rhabdo60 | 4 |
| Canu_Rhabdo61 | 2 |
| Canu_Rhabdo62 | 3 |
| Canu_Rhabdo63 | 4 |
| Canu_Rhabdo64 | 2 |
| Canu_Rhabdo65 | 1 |
| Canu_Rhabdo66 | 1 |
| Canu_Rhabdo67 | 1 |
| Canu_Rhabdo7 | 0 |
| Canu_Rhabdo8 | 1 |
| Canu_Rhabdo9 | 0 |
| Canu_Toti1 | 0 |
| Canu_Toti2 | 0 |
| Canu_Un1 | 0 |
| Canu_Virga1 | 5 |
| Canu_Virga2 | 2 |
| Canu_Virga3 | 0 |
| Canu_Virga4 | 2 |
| Canu_Xinmo1 | 1 |
| Canu_Xinmo2 | 0 |
| Canu_Xinmo3 | 0 |
| Canu_Xinmo4 | 1 |
| Canu_Xinmo5 | 0 |
| Canu_Xinmo6 | 0 |
| Canu_Mesoni1 | 4 |

**Table S7: PCR primers used to confirm newly identified viral integrations**

List of PCR primers used to confirm newly identified nrEVEs from wild-collected mosquitoes from Tampon and Tapachula.

| **nrEVE** | **Primer Sequence** | **F or R** | ***Location*** |
| --- | --- | --- | --- |
|  |  |  |  |
| nrEVEnew-1 | CATCAGCCTTTCCGTAGTTCC | F | nrEVE |
|  | CGACAGCCTGTTCGAATGC | R | *genome* |
|  |  |  |  |
| nrEVEnew-2 | CCGCGCTCTCACTCAGTA | F | *genome* |
|  | CCATCAGCACAAGATCATCAGT | R | nrEVE |
|  |  |  |  |
| nrEVEnew-3 | AAGTTCTCGCGACTAACCCA | R | nrEVE |
|  | GCCATCCAACTTGAACCGAT | F | *genome* |
|  |  |  |  |
| nrEVEnew-4 | CCGCGTTGGTCCCTTCTG | F | nrEVE |
|  | GTGAGTGCCCTATACGTTAGCA | R | *Genome* |

**Table S8: Expansion of PPO genes**

Data table listing genes in each PPO cluster and their position in the scaffold NW_021838576.1.

| **Cluster** | **Gene symbol** | **Gene** | **Merged_features** | **Location in NW_021838576.1** |
| --- | --- | --- | --- | --- |
| Cluster 1 | PPO1 | LOC109423858 | XM_019698895.2 and XP_019554440.2 | 52,804,654..52,815,218 |
|  | PPO2 | LOC109423857 | XM_019698893.2 and XP_019554438.2 | 52,784,310..52,795,491 |
|  | PPO4 | LOC109408601 | XM_029878549.1 and XP_029734409.1 | 52,769,025..52,778,400 |
|  | PPO5 | LOC115269861 | XM_029878648.1 and XP_029734508.1 | 52,719,809..52,749,080 |
|  | PPO7 | LOC109408587 | XM_019681910.2 and XP_019537455.1 | 52,709,465..52,711,860 (complement) |
|  | PPO9 | NA | NA | NA |
| Cluster 2 | PPO1 | LOC115253846 | XM_029879451.1 and XP_029735311.1 | 53,992,038..54,001,353 (complement) |
|  | PPO2 | NA | NA | NA |
|  | PPO4 | LOC115270202 | XM_029879455.1 and XP_029735315.1 | 54,037,682..54,050,189 (complement) |
|  | PPO5 | LOC109408583 | XM_019681907.2 and XP_019537452.2 | 54,058,612..54,061,155 (complement) |
|  | PPO7 | LOC115270200 | XM_029879453.1 and XP_029735313.1 | 54,076,259..54,078,936 (complement) |
|  | PPO9 | LOC115270199 | XM_029879452.1 and XP_029735312.1 | 54,106,216..54,108,834 |
| Cluster 3 | PP01 | NA | NA | NA |
|  | PPO2 | LOC115270203 | XM_029879456.1 and XP_029735316.1 | 54,212,535..54,217,346 (complement) |
|  | PPO4 | LOC109423856 | XM_019698892.2 and XP_019554437.2 | 54,223,957..54,229,104 (complement) |
|  | PPO5 | LOC115269863 | XM_029878652.1 and XP_029734512.1 | 54,244,769..54,281,901 (complement) |
|  | PPO7 | NA | NA | NA |
|  | PPO9 | LOC109423853 | XM_019698890.2 and XP_019554435.2 | 54,279,276..54,281,642 |
