# Supplementary material for: Improved reference genome of the arboviral vector Aedes albopictus
Source: Genome Biol. 2020 Aug 26;21:215. doi: 10.1186/s13059-020-02141-w (PMC7448346; doi:10.1186/s13059-020-02141-w)
Supplement: Supplementary file 12 — Additional file 12: Review history. [file 13059_2020_2141_MOESM12_ESM.docx]

Review History

**First round of review**

**Reviewer 1**

**Are you able to assess all statistics in the manuscript, including the appropriateness of statistical tests used?** Yes, and I have assessed the statistics in my report.

**Comments to author:**

This manuscript describes the development of a significantly improved reference genome assembly for Aedes albopictus, an important arbovirus vector. It is an important resource that will be of interest to many vector biologists. There are several innovative approaches described that will make the manuscript of interest to a wider audience, such as the use of cDNAs to perform physical mapping/anchoring of genome assembly scaffolds to chromosomes. The manuscript is well written, and the characterization of the assembly is accompanied by interesting exploratory biological analyses on a variety of topics, including population genomic resequencing of several populations samples. The manuscript could be improved through attention to a few minor points:

1) Page 5 (line numbering seems to be garbled in my PDF copy): Is it indeed still true that it is not possible to make sequencing libraries from single individual mosquitoes, or was that true at the time the data were made? I'm thinking of PMID: 30669388. If the Ae. albopictus genome is simply too large to generate sufficient coverage from a library made from a single individual mosquito, that could be helpful, but as this paper may serve as a guidepost for other groups attempting assembly for highly heterozygous arthropods, it could be useful to call out the Kingan paper and whether its approach is relevant in this context.

2) Page 18: need a unit for the Ae. aegypti r2 Max/2 (34-101 kb?) In figure 4E the scale of the horizontal axis is too coarse to actually see at what physical distance LD stops being above background in each population. Why does the x axis range go > 20 kb on that plot? The difference in LD between Ae. albopictus and aegypti is fascinating if true, but I am worried it is apples to oranges given the WGS vs SNP array basis. For example, how were SNPs chosen for the aegypti array? Are the SNPs enriched for high MAF, which could make them older than the SNPs profiled in albopictus? I realize this feature would bias aegypti towards exhibiting less LD than albopictus if true, but it's an example of the considerations required for this type of comparison.

3) Figure 2A: horiz axis should have units and/or some kind of label

4) Figure 4C: What should readers take away from this exactly? Why are the points in pileups with gaps between them? What is the significance of the result? Perhaps it would be more meaningful to calculate Fst for genes rather than for sliding windows.

5) Figure 4D: There should be labels on the horiz axis for chromosomes. The color scheme is different than in panels A and B. Shouldn't do a line graph with green and red as 5% of males will not be able to interpret.

6) FIgure 4E: The color scheme shifts again.

**Reviewer 2**

**Are you able to assess all statistics in the manuscript, including the appropriateness of statistical tests used?** There are no statistics in the manuscript.

**Comments to author:**

Review of "Improved reference genome of the arboviral vector Aedes albopictus" by Palatini et Al.

The authors present a significantly improved genome assembly, repeat and small RNA analysis and extensive transcriptome data for a critically important species that dramatically affects human and animal health. Because of the importance of the species to human health and the increasing risk of vectoring disease into previously unaffected areas due to climate change, I believe this work should have a higher profile publication in genome biology than for example BMC genomics.

The manuscript is generally well written, although perhaps a little wordy in some places, and the main text could be reduced a little to make it easier on the reader.

The quality of the new genome assembly is impressive with a contig N50 of 55Mbp a truly massive number for contiguity I have not seen in any other insect assembly - Drosophila chromosome arms are not this big. The authors are to be commended on doing an excellent job, despite having to deal with multiple alleles despite the sib-sib inbreeding, and not being able to use the newer single mosquito techniques which do not generate enough library and thus sequence yield to deal with a genome size this big. The busco stats look good, and I was particularly impressed by the work to identify and separate the remaining un-assembled gene alleles (described on p5), something that should be copied by many other groups. The physical map was also a nice touch. I was impressed with the methods for the assembly and I feel I would have no real trouble repeating the assembly of the raw dataset given the commands in the methods.

Data accessibility: I can easily find the genome assembly and raw read data in NCBI so that is excellent.

It was also nice to see a bit more of a traditional genome paper where foci of interest were deliberately curated and recorded for the reader. The new fashion of genome notes with no analysis because of the time and effort required too actually look at a genome is a significant problem as it reduces the value of genome sequencing for the community - which, for this species, is significant - and for the reader.

The analyses focused on RNA viral introgressions, piRNAs, miRNAs, immune related genes, the M determining loci which will be critical to understand for gene drive and possible male sterile release control methods. The expression analysis of the miRNAs is one of things that puts this manuscript out of BMC genomics and into genome biology, as it will be a much stronger and more cited resource in the future. The manual curation and analysis of this many immunity and non-coding genes is a significant effort that will not be copied in many communities but is necessary for highly accurate research in the future. Too many communities don't know one of their duplicated genes is not really there, or have a gap in the middle of an annotation. This kind of extensive manual curation of gene families of biological importance is critical for moving forward without a foundation of sand, and the authors are to be congratulated.
The analysis then finished with a small amount of population genomics, and a significant transcriptional data resource, that will be crucial for future researchers. This is the kind of thing that drosophila researchers are used to looking up in flybase whenever needed, but the rest of us in other species rarely have access to.


Overall this is an excellent new resource for a species critical to human health, and the manuscript will be well cited. The authors have added a lot of data to go beyond a boring genome paper, and I think it thus deserves the wider audience of genome biology rather than BMC genomics.

I have some minor issues with the text, but as I can see and use the resource directly from NCBI I see no reason to do more than accept with minor revisions.


Minor issues
Abstract, background,  - I'm not sure the genome is exceptionally complex, as I am not sure what that means, and compared to what - salamander? Redwood?. I think you have done a great job and don't need the hyperbole.

Abstract, conclusions,   - you have "up-to-date" twice. Perhaps the second one could be "dramatically improved"

P4 line 01 or 101: I don't think artificially duplicated alleles is correct, but rather alleles that failed to collapse in the assembly.
P4 line 108: could you put the number 2.54Gb of assembly size in context and explicitly tell the reader how close it is to what you expect.

P6 from "We developed a new mapping approach - to - orienting the genome scaffolds along the chromosomes" the text feels a bit method-y and redundant, and perhaps could be shortened and moved to methods. (unless the it is really a novel mapping approach, but I feel that people have been trying everything they can for probes for a long time. Also, how many alternate dyes were used?

P7 - The landscape of endogenous viral elements - this quite a long section, and it would be nice if it was made a little more compact for the reader. Also because of the length, a bit more on the importance in the first paragraph would aid the less specialized reader. I am not fully sure what is meant by "adaptive heritable immunity sequences" at the top of p9. I did like the population conclusion that came out at the end of this, and made if feel well worth reading.

Table S1, typo in "scaffold lenght" (sic)

**Authors Response**

**Point-by-point responses to the reviewers’ comments:**

Reviewer #1: This manuscript describes the development of a significantly improved reference genome assembly for *Aedes albopictus,* an important arbovirus vector. It is an important resource that will be of interest to many vector biologists. There are several innovative approaches described that will make the manuscript of interest to a wider audience, such as the use of cDNAs to perform physical mapping/anchoring of genome assembly scaffolds to chromosomes. The manuscript is well written, and the characterization of the assembly is accompanied by interesting exploratory biological analyses on a variety of topics, including population genomic resequencing of several populations samples. The manuscript could be improved through attention to a few minor points.

***REPLY: We greatly appreciate the reviewer’s interest in work.***

1) Page 5 (line numbering seems to be garbled in my PDF copy): Is it indeed still true that it is notpossible to make sequencing libraries from single individual mosquitoes, or was that true at the time the data were made? I'm thinking of PMID: 30669388. If the *Ae. albopictus* genome is simply too large to generate sufficient coverage from a library made from a single individual mosquito, that could be helpful, but as this paper may serve as a guidepost for other groups attempting assembly for highly heterozygous arthropods, it could be useful to call out the Kingan paper and whether its approach is relevant in this context.

***REPLY: Indeed, the Ae. albopictus genome size is still too large for the low-input prep described by Kingan et al., but we agree this method is a very promising development for the field of***

***arthropod genomics. We have revised the relevant text as follows and included the reference:***

***“Despite promising improvements to long-read sequencing methods that have enabled genome assembly from a single Anopheles coluzzii mosquito (Kingan 2019), the larger genome size of Aedes spp. mosquitoes (i.e. 1.2 Gb vs. 280 Mb for An. coluzzii) required pooling of heterozygous individuals and the necessity of removing haplotypic duplications prior to the creation of haploid reference scaffolds (9)”***

2) Page 18: need a unit for the *Ae. aegypti* r2 Max/2 (34-101 kb?) In figure 4E the scale of the horizontal axis is too coarse to actually see at what physical distance LD stops being above background in each population. Why does the x axis range go > 20 kb on that plot? The difference in LD between *Ae.* *albopictus* and *aegypti* is fascinating if true, but I am worried it is apples to oranges given the WGS vs SNP array basis. For example, how were SNPs chosen for the aegypti array? Are the SNPs enriched for high MAF, which could make them older than the SNPs profiled in albopictus? I realize this feature would bias aegypti towards exhibiting less LD than albopictus if true, but it's an example of the considerations required for this type of comparison.

***REPLY: We noticed that after few changes were added, the reference to the LD in now on page 24 and we added the “kb” unit value that was missing.***

***Regarding panel 4E, there is almost no difference between the LD estimates among the 3 populations with estimates around 1.3kb, this is the reason for the output of the panel. We are afraid we cannot change the image.***

***As the reviewer noticed that LD estimates are very small, and we are showing a plot that goes up to 100kb. We used the maximum of 100kb distance between SNPs to calculate LD (option –max_snp_dist 100 of the ngsLD package). The minimal distance recommended by the authors of the package is 50kb. Performing estimates with smaller values is not reliable. We changed the plot to show only 25kb but used the maximum of 100kb to perform LD estimates and model fitting.***

***Regarding the comparison of WGS data and SNP-chip, we did use a MAF filter of 0.1 for all datasets. We do agree that we have to take into consideration the different types of data that we used to estimate the LD, nevertheless we believe the striking difference between these species are biologically meaningful. We agree with the reviewer that calculating LD from WGS data or chip array may introduce biases because the average mutation age of the SNPs on the chip and the SNPs from WGS data may differ causing this LD difference between the two species. To address this possibility, we added a sentence in the main text on page 15 line 460 and including supporting references (REF. 59 and 60):***

***“The age of mutations can affect LD with younger mutations giving higher LD values, it is possible that SNP-chip data and WGS data differ in average age of mutations, as in the latter SNPs are estimated across the whole-genome with no or prior analyses”.***

3) Figure 2A: horiz axis should have units and/or some kind of label

***REPLY: Figure 2A was revised***

4) Figure 4C: What should readers take away from this exactly? Why are the points in pileups with gaps between them? What is the significance of the result? Perhaps it would be more meaningful to calculate Fst for genes rather than for sliding windows.

***REPLY: Given the number of populations we estimated Fst values from, we cannot comment more that observing that Fst varies across the genome with a few very high Fst values, especially on Chromosome 1.***

***We thank the reviewer for noticing the large gaps on the plot. We carefully checked the scripts and found a small problem that caused the presence of gaps, thus we re-run the pipeline and re-designed the plot. Overall results did not change.***

***Considering gene annotation was done automatically, with manual annotation only for immunity genes,***

***we reasoned calculating Fst by gene could be biased, instead calculating Fst for sliding windows treats the genome equally.***

5) Figure 4D: There should be labels on the horiz axis for chromosomes. The color scheme is different than in panels A and B. Shouldn't do a line graph with green and red as 5% of males will not be able to interpret.

6) FIgure 4E: The color scheme shifts again.

***REPLY: Figure 4 was revised in all its panels.***

Reviewer #2: Review of "Improved reference genome of the arboviral vector *Aedes albopictus"* by Palatini et al.

The authors present a significantly improved genome assembly, repeat and small RNA analysis and extensive transcriptome data for a critically important species that dramatically affects human and animal health. Because of the importance of the species to human health and the increasing risk of vectoring disease into previously unaffected areas due to climate change, I believe this work should have a higher profile publication in genome biology than for example BMC genomics.

The manuscript is generally well written, although perhaps a little wordy in some places, and the main text could be reduced a little to make it easier on the reader. The quality of the new genome assembly is impressive with a contig N50 of 55Mbp a truly massive number for contiguity I have not seen in any other insect assembly - Drosophila chromosome arms are not this big. The authors are to be commended on doing an excellent job, despite having to deal with multiple alleles despite the sib-sib inbreeding, and not being able to use the newer single mosquito techniques which do not generate enough library and thus sequence yield to deal with a genome size this big. The busco stats look good, and I was particularly impressed by the work to identify and separate the remaining un-assembled gene alleles (described on p5), something that should be copied by many other groups. The physical map was also a nice touch. I was impressed with the methods for the assembly and I feel I would have no real trouble repeating the assembly of the raw dataset given the commands in the methods.

Data accessibility: I can easily find the genome assembly and raw read data in NCBI so that is excellent. It was also nice to see a bit more of a traditional genome paper where foci of interest were deliberately curated and recorded for the reader. The new fashion of genome notes with no analysis because of the time and effort required too actually look at a genome is a significant problem as it reduces the value of genome sequencing for the community - which, for this species, is significant - and for the reader.

The analyses focused on RNA viral introgressions, piRNAs, miRNAs, immune related genes, the M determining loci which will be critical to understand for gene drive and possible male sterile release control methods. The expression analysis of the miRNAs is one of things that puts this manuscript out of BMC genomics and into genome biology, as it will be a much stronger and more cited resource in the future. The manual curation and analysis of this many immunity and non -coding genes is a significant effort that will not be copied in many communities but is necessary for highly accurate research in the future. Too many communities don't know one of their duplicated genes is not really there, or have a gap in the middle of an annotation. This kind of extensive manual curation of gene families of biological importance is critical for moving forward without a foundation of sand, and the authors are to be congratulated.

The analysis then finished with a small amount of population genomics, and a significant transcriptional data resource, that will be crucial for future researchers. This is the kind of thing that drosophila researchers are used to looking up in flybase whenever needed, but the rest of us in other species rarely have access to.

Overall this is an excellent new resource for a species critical to human health, and the manuscript will be well cited. The authors have added a lot of data to go beyond a boring genome paper, and I think it thus deserves the wider audience of genome biology rather than BMC genomics.

I have some minor issues with the text, but as I can see and use the resource directly from NCBI I see no reason to do more than accept with minor revisions.

***REPLY: We thank the reviewer for having so nicely outlined our work and recognized our efforts both in producing an accessible, usable and improved assembly and in our focused analyses of some aspects of the genome, which we think will be useful to many working on Ae. albopictus.***

Minor issues

Abstract, background, - I'm not sure the genome is exceptionally complex, as I am not sure what thatmeans, and compared to what - salamander? Redwood? I think you have done a great job and don't need the hyperbole.

Abstract, conclusions, - you have "up-to-date" twice. Perhaps the second one could be "dramatically improved"

***REPLY: Abstract was revised eliminating both “exceptionally complex” and the second “up-to-date”. Thank you for noticing the repetition.***

P4 line 01 or 101: I don't think artificially duplicated alleles is correct, but rather alleles that failed to collapse in the assembly.

***REPLY: “artificially duplicated alleles” was revised to “alleles that failed to collapse in the assembly”.***

P4 line 108: could you put the number 2.54Gb of assembly size in context and explicitly tell the reader how close it is to what you expect.

***REPLY: Using a cytofluorimetric approach, we estimated the haploid genome size to be roughly 1.25 Gb, a value which we report in the same paragraph (line 103).***

P6 from "We developed a new mapping approach - to - orienting the genome scaffolds along the chromosomes" the text feels a bit method-y and redundant, and perhaps could be shortened and moved to methods. (unless the it is really a novel mapping approach, but I feel that people have been trying everything they can for probes for a long time. Also, how many alternate dyes were used?

***REPLY: description of the methodology for in situ hybridization was eliminated from the result section. As indicated in Method section we used only two alternate dyes Cy3 and Cy5 to label the probe from the beginning and from the end of the scaffold. We may consider using additional label dyes for the mapping in the future.***

P7 - The landscape of endogenous viral elements - this quite a long section, and it would be nice if it was made a little more compact for the reader. Also because of the length, a bit more on the importance in the first paragraph would aid the less specialized reader. I am not fully sure what is meant by "adaptive heritable immunity sequences" at the top of p9. I did like the population conclusion that came out at the end of this, and made if feel well worth reading.

***REPLY: We shortened the paragraph on viral integrations to highlight novelties of this work with***

***respect to what previously published. We also mentioned novel exciting results on the biology of nrEVEs as sequences with antiviral activity and included the reference of the bioinformatic pipeline we developed to identify novel viral integrations (REF. 11, 12 and 17).***

Table S1, typo in "scaffold lenght" (sic)

***REPLY: corrected. Thank you for noticing.***
